# Integrating Statistical Predictions and Experimental Verifications for Enhancing Protein-Chemical Interaction Predictions in Virtual Screening

**DOI:** 10.1371/journal.pcbi.1000397

**Published:** 2009-06-05

**Authors:** Nobuyoshi Nagamine, Takayuki Shirakawa, Yusuke Minato, Kentaro Torii, Hiroki Kobayashi, Masaya Imoto, Yasubumi Sakakibara

**Affiliations:** Department of Biosciences and Informatics, Keio University, Yokohama, Japan; University of California San Diego, United States of America

## Abstract

Predictions of interactions between target proteins and potential leads are of great benefit in the drug discovery process. We present a comprehensively applicable statistical prediction method for interactions between any proteins and chemical compounds, which requires only protein sequence data and chemical structure data and utilizes the statistical learning method of support vector machines. In order to realize reasonable comprehensive predictions which can involve many false positives, we propose two approaches for reduction of false positives: (i) efficient use of multiple statistical prediction models in the framework of two-layer SVM and (ii) reasonable design of the negative data to construct statistical prediction models. In two-layer SVM, outputs produced by the first-layer SVM models, which are constructed with different negative samples and reflect different aspects of classifications, are utilized as inputs to the second-layer SVM. In order to design negative data which produce fewer false positive predictions, we iteratively construct SVM models or classification boundaries from positive and tentative negative samples and select additional negative sample candidates according to pre-determined rules. Moreover, in order to fully utilize the advantages of statistical learning methods, we propose a strategy to effectively feedback experimental results to computational predictions with consideration of biological effects of interest. We show the usefulness of our approach in predicting potential ligands binding to human androgen receptors from more than 19 million chemical compounds and verifying these predictions by in vitro binding. Moreover, we utilize this experimental validation as feedback to enhance subsequent computational predictions, and experimentally validate these predictions again. This efficient procedure of the iteration of the *in silico* prediction and in vitro or in vivo experimental verifications with the sufficient feedback enabled us to identify novel ligand candidates which were distant from known ligands in the chemical space.

## Author Summary

This work describes a statistical method that identifies chemical compounds binding to a target protein given the sequence of the target or distinguishes proteins to which a small molecule binds given the chemical structure of the molecule. As our method can be utilized for virtual screening that seeks for lead compounds in drug discovery, we showed the usefulness of our method in its application to the comprehensive prediction of ligands binding to human androgen receptors and in vitro experimental verification of its predictions. In contrast to most previous virtual screening studies which predict chemical compounds of interest mainly with 3D structure-based methods and experimentally verify them, we proposed a strategy to effectively feedback experimental results for subsequent predictions and applied the strategy to the second predictions followed by the second experimental verification. This feedback strategy makes full use of statistical learning methods and, in practical terms, gave a ligand candidate of interest that structurally differs from known drugs. We hope that this paper will encourage reevaluation of statistical learning methods in virtual screening and that the utilization of statistical methods with efficient feedback strategies will contribute to the acceleration of drug discovery.

## Introduction

In the early stages of the drug discovery process, prediction of the binding of a chemical compound to a specific protein can be of great benefit in the identification of lead compounds (candidates for a new drug). Moreover, the effective screening of potential drug candidates at an early stage generates large cost savings at a later stage of the overall drug discovery process.

In the field of virtual screening for the drug discovery, docking analyses and molecular dynamics simulations have been the principal methods used for elucidating the interactions between proteins and small molecules [1]–[4]. Fast and accurate statistical prediction methods for binding affinities of any pair of a protein and a ligand have also been proposed for the case where information regarding 3D structures, binding pockets and binding affinities (e.g. pK*i*) for a sufficient number of pairs of proteins and chemical compounds is available [5]. However, the requirement of these programs for 3D structural information is a severe disadvantage, as the availability of these data is extremely limited. Although a number of structures in PDB [6] is increasing (from 23,642 structures in 2003 to 48,091 structures in 2007), not all proteins which have been derived from many genome-sequencing projects are suitable for experimental structure determination. Hence, the genome-wide application of these methods is in fact not feasible. For example, among the GPCRs (G-protein coupled receptors), whose modulation underlies the actions of 30% of the best-known commercial drugs [7], the full structure of only a few mammalian members, including bovine rhodopsin [8] and human beta 2 adrenoreceptor [9], is known.

To achieve more comprehensive and faster protein-chemical interaction predictions in the post-genome era producing a vast number of protein sequences whose structural information is not available, it is essential to be able to utilize more readily available biological data and more generally applicable methods which do not require 3D structural data [10]–[12]. In our previous study, we developed a comprehensively applicable statistical method for predicting the interactions between proteins and chemical compounds by exploiting very general biological data, including amino acid sequences, 2-dimensional chemical structures, and mass-spectrometry (MS) data [11]. These statistical approaches provided a novel framework where the input space consists of pairs of proteins and chemical compounds. These pairs are classified into binding and non-binding pairs, while most chemoinformatics approaches assess only chemical compounds and classify them according to their pharmacological effects. Our previous study [11] demonstrated that screening target proteins for a chemical compound could be performed on a genome-wide scale. This is due to the fact that our method can be applied to all proteins whose amino acid sequences have been determined even though the 3D structural data is not yet available. Genome-wide target protein predictions were conducted for MDMA, or ecstasy, which is one of the best known psychoactive drugs, from a pool of 13,487 human proteins, and known bindings of MDMA were correctly predicted [11].

Although the method yielded a relatively high prediction performance (more than 80% accuracy) in cross-validation and usefulness in the comprehensive prediction of target proteins for a given chemical compound with tens of thousands of prediction targets [11], it suffered from the problem of predicting many false positives when comprehensive predictions were conducted. Although these false positives might include some unknown true positives, they were mainly due to the low quality of the negative data, which is one of the common problems in utilizing statistical classification methods such as Support Vector Machines (SVMs) and Artificial Neural Networks (ANNs).

In this paper, we describe two strategies, namely two-layer SVM and reasonable negative data design, which are used for the purpose of reducing the number of false positives and improving the applicability of our method for comprehensive prediction. In two-layer SVM, in which outputs produced by the first-layer SVM model are utilized as inputs to the second-layer SVM, in order to design negative data which produce fewer false positives, we iteratively constructed SVM models or classification boundaries and selected negative sample candidates according to pre-determined rules. By using these two strategies, the number of predicted candidates was reduced to around 100 (Table 1) in experiments in which the potential ligands for some druggable proteins (UniProt ID P10275 (androgen receptor), P11229 (muscarinic acetylcholine receptor M1) and P35367 (histamine H1 receptor)) are predicted on the basis of more than 100,000 compounds in the PubChem Compound database (http://pubchem.ncbi.nlm.nih.gov/).

**Table 1:** Evaluation of our method with respect to comprehensive interaction prediction.

dataset^1^	neg.^2^	1sts^3^	P10275^4^	P11229^4^	P35367^4^	rec_0.5_ (%)^5^	rec_0.95_ (%)^5^	evaluation^6^
(A)								
*mlt*	16	–	714	1408	1187	100	98.97	82.50
*random*	16	–	1869.3(±136.1)	10503.3(±1250.7)	9305.3(±517.8)	100	99.66(±1.09)	69.45(±0.32)
(B)								
*mlt*	14	10	177	535	451	96.91	93.81	75.56
*random*	14	10	848.3(±345.0)	1531.7(±628.9)	988.0(±411.4)	96.56(±2.89)	81.10(±19.44)	66.44(±7.82)
(C)								
*max*	16	9	28	231	129	100	97.94	82.92
*random*	16	9	74.7(±42.6)	255.3(±32.2)	146.7(±8.3)	100	100	80.67(±0.93)
(D)								
–	–	–	640	1791	838	86.60	71.13	59.66
(E)								
–	–	–	1869	1816	1580	–	–	–

(A) One-layer SVM. (B) Two-layer SVM with the first-layer SVM models based on the *subpos* datasets. (C) Two-layer SVM with the first-layer SVM models based on the *allpos* datasets. (D) ^†^SVM only utilizing chemical compound information. (E) ^‡^Similarity search.

**†:** SVM model which only classifies chemical compounds (not pairs) according to the binding property to the target proteins. Chemical compounds binding to each target protein were treated as positives, and all other compounds in the DrugBank dataset were regarded as negatives.

**‡:** A chemical compound *i* was predicted as binding to a protein α by using the similarity method if 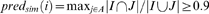, where *A* represents the known binding ligands of α, and *I* (or *J*) represents a set of substructures considered in calculating the feature vector of the chemical compounds.

1 refers to negative data expansion rules (details are provided in Materials and Methods). “random” indicates that three types of random pairs comprising a protein and a drug are used as negatives. The 95% confidence intervals are shown.

2 the number of negatives ( = 1,750×*x*).

3 the number of first-layer SVM models utilized to construct the second-layer SVM model.

4 target proteins whose ligands were predicted from 109,841 compounds. The number of predicted ligands is shown.

5 rec*_x_* is the recall rate( = *TP*/(*TP*+*FN*)) at the threshold *x*. 0.5 is the threshold following the definition of SVM. *TP*: true positives, *FN*: false negatives.

6

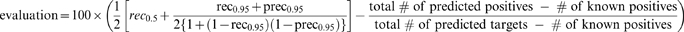
(1) Here, prec*_x_* is the precision ( = *TP*/(*TP*+*FP*)) at the threshold *x*. *FP*: false positives.

With the aim of validating the usefulness of our method, our proposed prediction model with fewer false positives was applied to the PubChem Compound database in order to predict the potential ligands for the “androgen receptor”, which is one of the genes responsible for prostate cancer. We verified some of these predictions by measuring the IC_50_ values in an in vitro assay.

Biological experiments, conducted to verify the computational predictions based on statistical methods, docking methods or molecular dynamics methods, typically involve success as well as failure. In addition to fast calculation and wide applicability, one of the merits of using statistical methods that involve training with known data is that results obtained by verification experiments can be efficiently utilized as feedback to produce new and more reliable predictions. Most previous work on virtual screening has focused on the computational prediction and listing of dozens or hundreds of candidates, followed by their experimental verification. However, only on rare occasions have these experimental results been utilized for the further improvement of computational predictions and experiments. Moreover, even without verification experiments, additional data acquired from, for example, relevant literature can be used for enhancing the prediction reliability.

**Figure 1:**
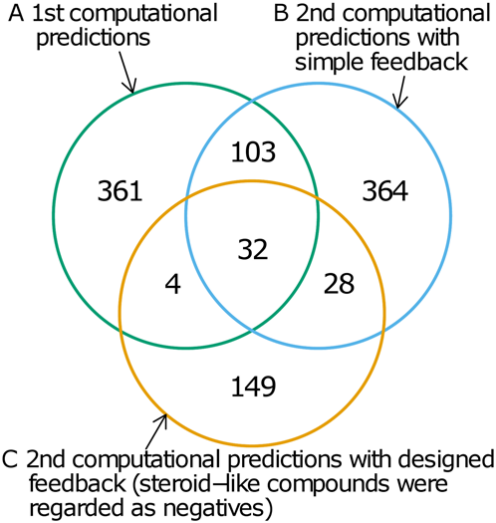
The scope of the predictions changed depending on whether feedback data were used and how they were utilized. (A) 500 predictions without feedback data. (B) 527 predictions with feedback from the first experimental verification. (C) 213 predictions based on the feedback strategy where pairs of chemical compounds with steroid structures and the androgen receptor were regarded as negatives.

**Figure 2:**
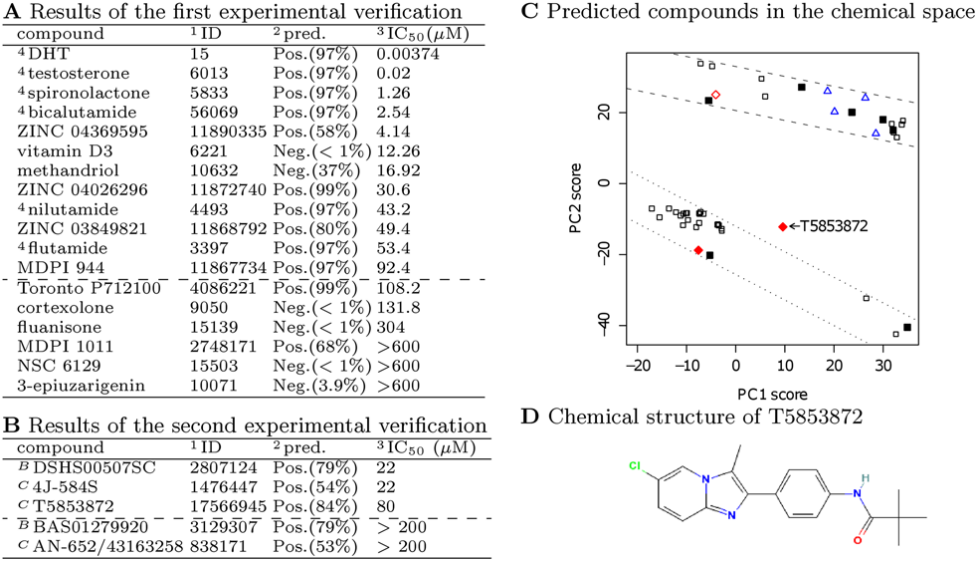
The first and the second experimental verifications showed more than 60% accuracy of computational predictions and the chemical space of verified compounds was explored. (A) Results of the first in vitro binding assay. (B) Results of the second in vitro binding assay. (C) The chemical space based on E-Dragon [14] descriptors and the principal component analysis (PCA) applied to known ligands and additional data. The black squares correspond to known ligands in the training data, the solid black squares represent known approved drugs, the blue triangles correspond to true positives in the first computational prediction, and the red diamonds correspond to true positives in the second computational prediction. The open red diamonds belong to Figure 1B, and the solid red diamonds belong to Figure 1C. Chemical compounds located between the two dashed lines have steroid-like structures. (D) A potential ligand with a chemical structure differing from the structures of known ligands. In (A) and (B), ^1^; PubChem Compound ID. ^2^; computational prediction expressed as “label (predicted probability for a positive outcome)”. ^3^; The concentration of an unlabeled test compound, in which, according to the measured radioactivity, 50% of the [^3^H]-DHT is still bound to MBP-ARC. ^4^; chemical compounds included in the DrugBank set or additional data. *^B^* ^(*C*)^; predictions belonging to Figure 1B (C).

Therefore, we propose a strategy based on the effective combination of computational prediction and experimental verification. Our second computational prediction utilizing feedback from the first experimental verification successfully discovered novel ligands (Figure 1 and 2) for the androgen receptor. Our approach suggests the significance of utilizing statistical learning methods and feedback from experimental results in drug lead discovery.

In the following section, we first describe the real application of our method involving the computational prediction, the experimental verification and the feedback, and then explain the computational experiments conducted to verify the usefulness of our computational prediction method in comprehensive prediction.

## Results

### Application of our strategy to the discovery of androgen receptor binding ligands

#### First computational prediction

We set the human androgen receptor (AR) as the target protein, whose binding ligands were predicted by using the PubChem database. Here, AR is a steroid hormone receptor and a transcription factor belonging to the nuclear receptor superfamily. In pathology, AR is one of the genes responsible for prostate cancer, which is the most frequently diagnosed cancer in men in the United States according to the American Cancer Society Statistics for 2008. The two-layer SVM model with an additional model for the androgen receptor, which constitutes a prediction model trained on the basis of supplementary information obtained from the relevant literature or databases as well as feedback from experimental verifications, was applied to the screening for human androgen receptor binding ligands from 19,171,127 chemical compounds in the PubChem Compound database. As a result, 500 chemical compounds (compounds with the same connectivity were counted only once) were predicted (Figure 1A).

#### First experimental verification

Out of 500 computationally predicted candidates, an in vitro binding assay was applied to 18 purchasable chemical compounds (details are provided in Figure S4), which were chosen by considering chemical structures and predicted probabilities from 43 chemical compounds marked as purchasable in ChemCupid (http://www.namiki-s.co.jp/chemcupid/) in October 2007, and there were 6 known drugs or androgens among the chosen chemical compounds (Figure 2A). The results obtained for these 6 known ligands agreed well with the results found in the relevant literature [13], thus proving the reliability of the assay.

For 12 predictions, except 6 known ligands, by applying a threshold level of IC_50_ = 100 µM, which was based on the fact that IC_50_ of flutamide was more than 50 µM, a precision of 67% (4/6) and an accuracy of 67% (8/12) were obtained (Figure 2A). As a result, it was possible to subsequently refine the predictions by using two misclassified compounds which were not detected in our method but which proved to bind to the androgen receptor.

#### Second computational prediction with feedback

By utilizing the results of the first experimental verification, the prediction model was reconstructed. Although the first computational prediction and experimental verification involved many compounds with steroid skeletons, binding of steroid-like compounds to the androgen receptor, which is a steroid-hormone receptor, is relatively obvious. Moreover, since steroid-like compounds are expected to act as agonists of the androgen receptor, antagonists are given preference in terms of search for chemical compounds with potential therapeutic effects for human prostate cancer, which involves activation of the androgen receptor. Thus, the prediction model in which pairs of the androgen receptor and steroid-like chemical compounds were regarded as negatives was also constructed in order to search for antagonists of the androgen receptor. The prediction coverage of these two models (Figure 1B and 1C) was different. The latter prediction models predicted chemical compounds without steroid skeletons, as expected.

#### Second experimental verification

Among the second predictions, experimental verification was performed with respect to 5 purchasable candidates, which were predicted with the two models reconstructed with feedback data and different strategies, as described in the previous section, and which were selected from predictions specific to each model, including 49 compounds marked as purchasable in ChemCupid in July 2008 (details are provided in Figure S4). Out of these 5 candidates, 3 chemical compounds bound to the androgen receptor at a threshold of 100 µM (Figure 2B), thus achieving 60% precision (3/5).

As shown in Figure 2C, known drugs and chemical compounds in the additional data can be roughly divided into two regions in the chemical space, which is based on the results of the Principal Component Analysis (PCA) applied to known ligands and chemical compounds in additional data represented by E-Dragon descriptors [14]. Although all true positives of the first computational prediction belonged to one of these regions, T5853872 (Figure 2C and 2D), which is one of the second computational predictions based on the designed strategy, was not included in these regions. This result suggests that repeating the processes of the computational prediction, the experimental verification and the feedback of the experimental results for new predictions contributes to the efficient exploration of the chemical space targeted in the search as well as to the discovery of novel ligands.

The third computational prediction, which utilized the results of the second experimental verification, further extended the predictions (details are provided in Text S1 and Figure S5) and successfully predicted chemical compounds which were of structural variety (data not shown). The repetition of the process of integrating computational prediction and experimental verification continues to provide novel candidates.

### Indication of the biological validity of statistical approaches

In bioinformatics, statistical approaches extract rules from numerical data corresponding to biological properties. Here, it is not guaranteed that the extracted rules are biologically valid, and furthermore it is possible to utilize statistical methods to obtain general rules from any kind of numerical data which are meaningless and irrelevant to biological properties. The biological relevance of our approach can be verified as follows on the basis of supporting evidence which indicates that our method can extract significant rules only if biologically valid and relevant data is given.

First, high prediction performances on diverse datasets might support the validity of our approach. In several datasets consisting of known pairs of proteins, including nuclear receptors, GPCRs, ion channels and enzymes, and drugs and random protein-drug pairs, our statistical approach with SVM showed high prediction performances (details are provided in Text S1, Table S1 and Figure S2). The fact that more than 0.85 AUC and an accuracy of 80% were obtained for diverse datasets suggests that it is possible to extract some properties accountable for interactions between proteins and drugs by statistical approaches. This possibility can be further supported by the fact that integrating several datasets whose target proteins were not relevant to each other improved the prediction performances with respect to pairs of proteins and chemical compounds which had a specific binding mode (details are provided in Text S1 and Table S2).

**Figure 3:**
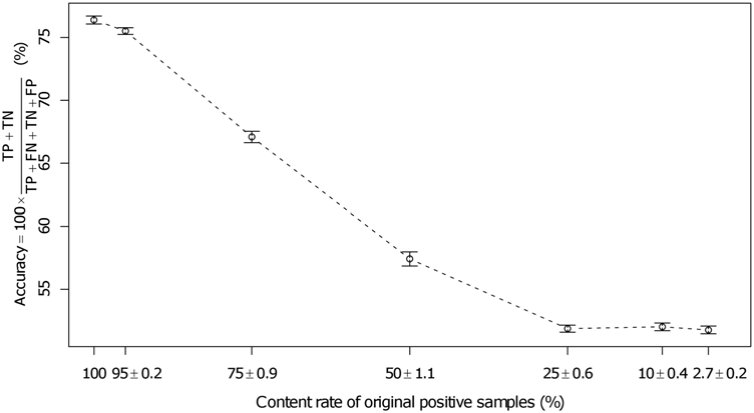
The prediction accuracy is proportional to the content rate of biologically valid samples. The average of 10 datasets produced by shuffling pairs corresponded to each content rate (ex. 50%) of pairs comprising a protein and a chemical compound in the original dataset. A usual SVM training, which is referred to as the first-layer SVM in the Materials and Methods section, and a 10-fold cross-validation evaluation were performed for each dataset of 1,731 positives and 1,750 negatives (or random pairs other than positives). Here, the SVM parameters were selected in such a way that they gave the best accuracy.

Second, we showed the biological relevance of these high prediction performances by calculating the prediction performances using biologically meaningless artificial datasets as positives. Several datasets which contained fractions of valid samples found in the DrugBank dataset, and which comprised artificial pseudo-positive samples of protein-chemical pairs produced by shuffling with the same frequency of chemical compounds and proteins as that in the DrugBank dataset, were generated. Our method was applied to these shuffled artificial datasets (Figure 3). Here, if our approach did not depend on the biological properties of the given dataset but only succeeded in classifying given pairs comprising a protein and a chemical compound and random pairs derived from them, the prediction accuracy for each shuffled dataset was assumed not to fluctuate.

As shown in Figure 3, the prediction accuracy was proportional to the content rate of the biologically valid samples. Therefore, the classification of our approach was shown to function only when a certain amount of biologically valid pairs comprising a protein and a chemical compound are given. This result suggests that our statistical approach succeeds in extracting the rules which are only relevant for the biological binding properties.

### False positive reduction in comprehensive prediction

It is often observed that although statistical learning approaches achieve very high prediction performances in given datasets, statistical prediction models suffer from the problem of generating vast prediction sets including many false positives when applied to a huge dataset, such as the PubChem database. In our approach, SVM models based on feature vectors directly representing amino acid sequences, chemical structures, and random protein-compound pairs as negatives also produced many predictions and inevitably yielded many false positives (Table 1A *random*).

Upon the introduction of the two-layer SVM and the negatives designed to overcome this drawback, the prediction precision, or the confidence of positive prediction, was significantly improved in computational experiments based on the DrugBank dataset (Table 2). In Table 2, the external dataset consisted of 170 positives and 2,450 negatives that were randomly chosen from 1,731 positives and 24,500 designed negatives with the *mlt* rule (details are provided in Materials and Methods) and that were excluded in constructing first-layer and second-layer SVM models. The external dataset contained much more negatives than positives as it simulated the real application of virtual screening with vast databases where only a fraction of chemical compounds in the databases have the effect of interest. Tables 2A and 2B showed improvement of precision by introducing the designed negatives and the two-layer SVM respectively. Table 2B also indicated that the application of SVM to outputs of the first-layer SVM models was superior to other statistical learning methods [15] and naive combination of the first-layer SVM models, and that rational selection of the first-layer SVM models achieved significant higher precision (*P*-value = 0.0081 by *t* test) than randomly selected models (other comparisons are provided in Text S1, Table S3 and Table S4). Particularly, the second-layer SVM utilizing the *allpos* first-layer SVM models achieved higher precision than use of higher thresholds in the other SVM models (Table 2C). The high precision contributes to the selection of more reliable predictions and thus to the reduction of the number of false positives.

**Table 2:** Evaluation of our method with respect to internal and external prediction of the dataset.

Model type†	prec.*^in^* (%)‡	sens.*^in^* (%)‡	acc.*^in^* (%)‡	prec.*^ex^* (%)‡	sens.*^ex^* (%)‡	acc.*^ex^* (%)‡
(A)						
one-layer(designed)	71.76	42.99	95.11	64.66	50.59	95.00
one-layer(random)	82.38(±0.64)	38.22(±0.95)	95.38(±0.06)	40.68(±1.19)	50.00(±1.87)	92.02(±0.28)
(B)						
*subpos*	97.11	92.57	99.33	82.81	31.18	95.11
*subpos*(r.f.)	95.66(±0.32)	78.33(±1.60)	98.33(±0.10)	78.76(±2.86)	25.59(±1.09)	94.71(±0.09)
*voting*	-	-	-	8.89	57.06	59.27
*2^nd^ ANN*	95.98	93.21	99.29	75.81	27.65	94.73
*2^nd^ QDA*	70.69	54.39	95.49	34.52	17.06	92.52
(C)						
*allpos*	99.68	100.00	99.98	100.00	10.59	94.20
*subpos*(*t* = 0.9)	-	-	-	90.70	22.94	94.85
one-layer(*t* = 0.9)	-	-	-	86.67	15.29	94.35

(A) Effect of rational negative design. (B) Effect of the second-layer SVM with designed negatives. (C) Improvement of precision with the two-layer SVM ant the type of the first-layer SVM models.

**†:** “Model type” exhibits the one-layer SVM model or the second-layer SVM, which is specified by the type of 11 first-layer SVM model, was utilized. Here,

• (designed) means that the rationally designed negatives was used to construct the SVM model.

• (random) means that three types of randomly chosen 22,050 pairs of protein and chemical compounds were used use to construct the SVM model. The 95% confidence intervals were shown.

• (r.f.) means that twenty types of randomly chosen 11 first-layer SVM models were used to construct the second-layer SVM model.

• *2^nd^ ANN* means that Artificial Neural Network (ANN; implemented by the statistical software package R (http://cran.r-project.org/) function *nnet* [15]) was applied to outputs of 11 *subpos* first-layer SVM models. Parameters were selected to give the best accuracy in internal 10-fold cross validation. For example, 17 units were used in the hidden layer.

• *voting* means that voting with 11 *subpos* first-layer SVM models was used for prediction.

• *2^nd^ QDA* means that Quadratic Discriminant Analysis (QDA) (implemented by R function *qda* [15]) was applied to outputs of 11 subpos first-layer SVM models.

• (*t* = 0.9) means that final probability outputs were evaluated with the threshold *t* = 0.9.

**‡:** precision (prec.) = *TP*/(*TP*+*FP*), sensitivity (sens.) = *TP*/(*TP*+*FN*), accuracy (acc.) = (*TP*+*TN*)/(*TP*+*FN*+*TN*+*FP*). *TN*: true negatives. Here,

• *^ex^* means the prediction performances of the external prediction. The external dataset consisted of 170 positives and 2,450 negatives that were randomly chosen from 1,731 positives and 24,500 designed negatives with the *mlt* rule (details are provided in Materials and Methods) and that were excluded in constructing first-layer and second-layer SVM models.

• *^in^* means the prediction performances of internal 10-fold cross-validation. The internal dataset utilized 1,561 positives and 22,050 negatives, which were not included in the external dataset.

Following these results on given datasets, our approaches were evaluated with respect to comprehensive binding ligand prediction. For three proteins (UniProt ID P10275 (androgen receptor), P11299 (muscarinic acetylcholine receptor M1) and P35367 (histamine H1 receptor)), their binding ligands were predicted from PubChem Compound 0000001–00125000 which contains 109,841 compounds (Table 1). Here, P35367 and P11299 are the two most frequently targeted proteins in the DrugBank dataset, and P10275 is a protein of average occurrence in the DrugBank dataset. Among the 109,841 compounds, 47, 45, and 5 known ligands were included for P35367, P11299, and P10275, respectively.

As shown in Tables 1A, 1B and 1C, the use of carefully selected negatives, the introduction of the two-layer SVM, and the integration of these two approaches efficiently reduced the number of predictions and thus the number of false positives. For example, in comparison to Tables 1A and 1C, the number of candidates discovered by using the *max* dataset in the *allpos* two-layer SVM approach was about one fiftieth of the number of chemical compounds predicted by using the *random* negative dataset in the one-layer SVM. Furthermore, in comparison to other approaches based solely on the use of chemical compounds (Tables 1D and 1E), our approaches gave a reasonable number of predictions (other comparisons are described in Text S1 and Tables S5, S6, S7).

These results suggest that our prediction models select a reasonable number of ligand candidates from all chemical compounds in large databases and encourage the comprehensive binding ligand prediction for the target protein.

### Utilization of feedback and additional data

The experimental verification of the computational predictions produces feedback data or samples which are not included in the given training datasets. The efficient utilization of these data can contribute to the fast identification of compounds with the desired properties and can be of advantage to statistical learning approaches.

We compared several strategies for utilizing feedback data as follows. For three proteins (UniProt ID P10275 (androgen receptor), P11299 (muscarinic acetylcholine receptor M1) and P353367 (histamine H1 receptor)), ligand data which were not included in the DrugBank dataset were collected from relevant literature [16]–[18] and public databases, PDSP Ki database [19] and GLIDA [20], in February 2008. Overall, 35 androgen receptor-ligand pairs, 49 muscarinic acetylcholine receptor M1-ligand pairs, and 1,060 histamine H1 receptor-ligand pairs were supplemented. Additional models were constructed by using these supplemental pairs as positives (details are provided in Text S1).

**Figure 4:**
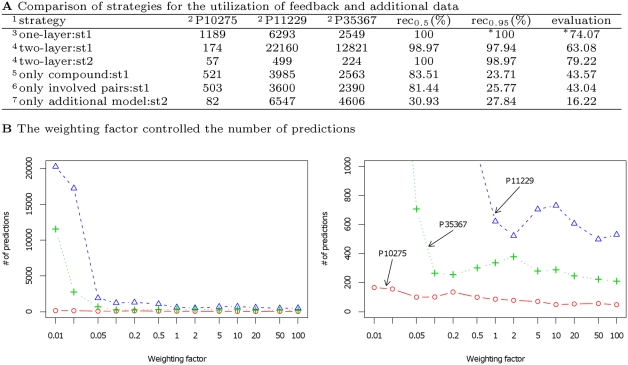
Effects of the strategy for the utilization of feedback and additional data. (A) ^1^: st1; a strategy where additional data, or pairs comprising a chemical compound and a protein, were simply added to the training samples in constructing a prediction model. st2; a strategy where additional data were first used for the construction of an additional first-layer SVM model and subsequently added to the training samples in the construction of a second-layer SVM model. ^2^: target proteins whose ligands were predicted from 109,841 compounds. The number of predicted ligands is shown. ^3^: one-layer SVM using the *mlt* dataset with 28,000 negatives. ^4^: two-layer SVM using 9 *allpos* first-layer SVM models and the *max* dataset with 28,000 negatives. In st2, the weighting factor was set to 50. ^5^: SVM model where the chemical compounds binding to each target protein were treated as positives, and all other compounds in the DrugBank dataset were regarded as negatives. ^6^: SVM model where pairs of all target proteins and known ligands were treated as positives, while pairs of all target proteins with other compounds were regarded as negatives. ^7^: two-layer SVM model in which only one first-layer SVM model derived from additional data was used for the construction of a second-layer SVM model. ^*^: a threshold of 0.9 was used instead of 0.95 for the calculation of “evaluation” (Eq. (1)). (B) The relation between the weighting factors and the number of predictions is shown for the case where the threshold = 0.5.

As shown in Figure 4, the use of the additional model with a sufficient weighting factor controlled the increase of the predictions with a slight decrease of the recall rate. The use of large weighting factors results in the relative decrease of the influence of other first-layer SVM models derived from the DrugBank dataset in classification. However, the low performance of “only additional model:st2”, shown in Figure 4A, where only one first-layer SVM model derived from additional data was used to construct the second-layer SVM model, indicates the need for first-layer SVM models derived from the DrugBank dataset as well as combinations of these first-layer SVM models with an additional first-layer SVM model.

With this efficient strategy for utilizing feedback data, computational prediction and experimental verification improve each other to enable faster search toward the identification of useful small molecules.

## Discussion

We proposed a comprehensively applicable computational method for predicting the interactions between proteins and chemical compounds, in which the number of false positives was reduced in comparison to other methods. Furthermore, we proposed the strategy for the efficient utilization of experimental feedback and the integration of computational prediction and experimental verification.

The application of our method to the androgen receptor resulted in 67% (4/6) prediction precision according to in vitro experimental verification in the first computational prediction and 60% (3/5) in the second prediction, which included the feedback of the first experimental verification. However, these relatively low precision values do not represent the true statistical significance of the method.

This 60–70% precision can also be evaluated by using the following *P*-value.
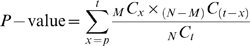
Here, *N* is the number of prediction targets, *M* the number of ligands potentially binding to the target proteins, *t* is the number of tested compounds, and *p* is the number of true positives. With *N* = 19171127, which is the number of chemical compounds in the PubChem Compound database, and *M* = 19171127×(456/3000)×(7/964)≒21160, which is based on the optimistic assumption that all compounds can be regarded as potential drugs for some target protein, it is estimated that 3,000 druggable proteins exist [21]. Moreover, the distribution of target proteins and drugs in the DrugBank dataset, consisting of 456 target proteins and 964 drugs, including 7 known ligands for the human androgen receptor, and *P*-values of 2∶21×10^−11^ and 1∶34×10^−8^ are obtained for the prediction precision of the first and the second computational prediction, respectively. These extremely small *P*-values prove the significance of the virtual screening and its precision in the drug discovery process.

**Figure 5:**
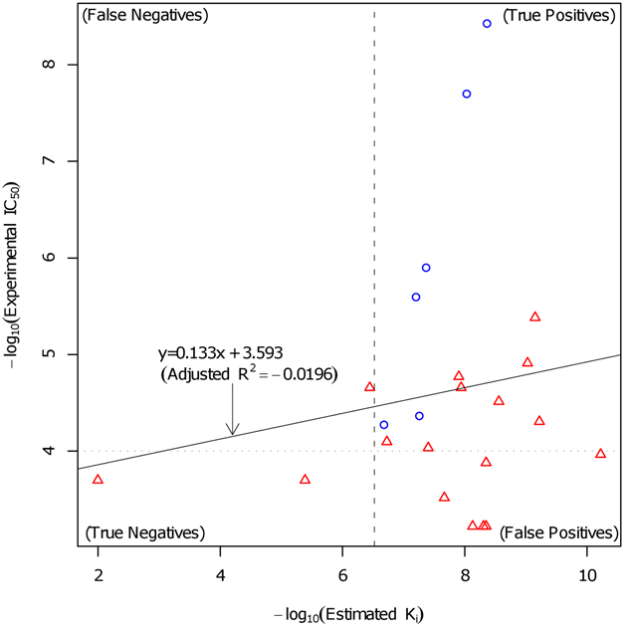
Docking analyses of experimentally verified chemical compounds. The blue circles denote known compounds and the red triangles denote other tested compounds. 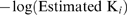 was derived from the estimated inhibition constant of the first cluster in the AutoDock output. Also, the horizontal dotted line denotes the threshold of 100 µM and the vertical dashed line denotes the threshold of 300 nM, which is based on the estimated K*_i_* 210.27 nM of flutamide, a known drug. With this threshold, 59% accuracy (10/17) and 57\% precision (8/14) were achieved while our method obtained an overall 65% accuracy (11/17; 8/12 in the first experimental verification and 3/5 in the second experimental verification) and 64% precision (7/11; 4/6 in the first experimental verification and 3/5 in the second experimental verification).

These prediction performances are as good as or better than several previous virtual screening studies based mainly on docking analyses [22]–[24]. For example, at a threshold of 100 µM, 7% precision (3/39) for *Mycobacterium tuberculosis* adenosine 5′-phosphosulfate reductase [22], 71% precision (22/31) for *Staphylococcus aureus* methyonyl-tRNA synthetase [23] and 8% precision (16/192) for human DNA ligase I [24] were obtained, respectively. In addition, 0.566 AUC was achieved in the docking analysis using AutoDock [3] (Figure 5) for the 17 chemical compounds (12 chemical compounds verified in the first experimental verification, with the exception of 6 known drugs, and 5 chemical compounds verified in the second experimental verification). In contrast, 0.681 AUC was obtained with our method. Here, in the calculation of AUC, the threshold level of IC_50_ = 100 µM for experimental verification was used to define a label (binding or non-binding) for each chemical compound, and 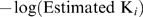 or the predicted probability was regarded as a value for each molecule. Note that the docking analysis with AutoDock was not applied to the 19,171,127 compounds in the PubChem Compound database for the screening purpose, but was applied only to 17 compounds, which were the results of virtual screening by our method. In terms of computational time, for binding prediction of one pair of a protein and a chemical compound, using one Opteron 275 2.2 GHz CPU, AutoDock took approximately 100 minutes on average with 100 genetic algorithm (GA) runs, while our method required less than 0.3 seconds. These computational time comparisons indicate that our method can perform a virtual screening of more than 19 million chemical compounds from the PubChem Compound database for any proteins in genome-wide scale and this immense screening task would be infeasible to accomplish with any of the existing docking methods. Therefore, our statistical approach can contribute as the first fast and rather accurate virtual screening tool for the drug discovery process. It can be followed by the application of more time-consuming but more informative approaches, such as docking analysis and molecular dynamics analysis, which can provide information regarding the binding affinities and the molecular binding mechanisms to outputs of the first screening.

**Figure 6:**
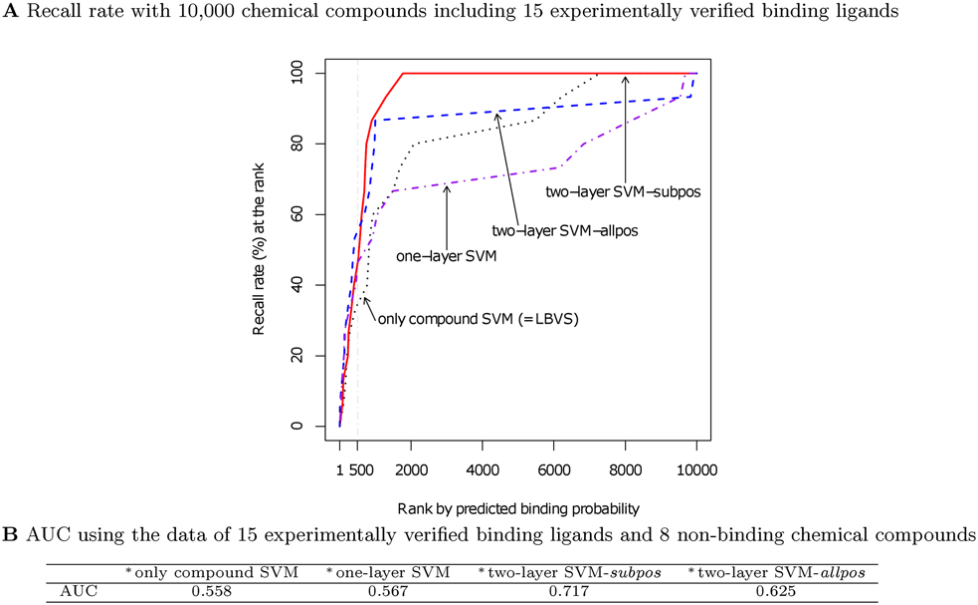
Re-evaluation of our method using the data of experimentally verified chemical compounds. (A) Evaluation by recall rate with 10,000 chemical compounds. Here, the recall rate at the rank *x* in descending order of predicted binding probability was calculated as (the number of 15 binding ligands whose rank is higher than *x*)/15. The 10,000 tested chemical compounds included 1,041 predicted ligand candidates, as shown in Figure 1, and 8,959 of the compounds were found within PubChem Compound CID 1-10427. (B) AUC using the data of 15 experimentally verified ligands and 8 non-binding chemical compounds. ^*^: In both (A) and (B), the prediction models were constructed as described in Figure 4, where 6 known chemical compound-androgen receptor pairs or 6 known chemical compounds among the 23 verified chemical compounds were excluded from the dataset utilized to construct the final prediction model and the weighting factor for two-layer SVM models was set to 10.

In another perspective, the re-evaluation of statistical prediction approaches by using 23 chemical compounds experimentally verified in this study showed that our proposed methods, which utilized information of both protein sequence and chemical structures, were superior to a conventional LBVS (Ligand Based Virtual Screening) method where only structures of specific chemical compounds were considered (Figure 6). As shown in Figure 6A, our proposed methods (“one-layer SVM”, “two-layer SVM-*subpos*” and “two-layer SVM-*allpos*”) achieved a higher recall rate at ranks higher than 500 compared to a conventional Ligand Based Virtual Screening method (“only compound SVM” in Figure 6A). The fact that experimentally verified chemical compounds were identified at higher ranks in the pool by our proposed prediction models suggests that our proposed models were highly efficient with respect to the screening method. Figure 6B also shows that our proposed methods were more successful at discriminating between 15 experimentally verified binding and 8 non-binding ligands better than the LBVS method. These comparisons suggest that our proposed method utilizing information of protein sequences as well as chemical structures can be regarded as a more useful substitute for usual ligand-based virtual screening methods utilizing only chemical structures.

Furthermore, the fact that the second computational prediction, or the use of feedback data, contributed to the discovery of novel ligands (Figure 2B–D) supports the utilization of statistical learning methods in virtual screening.

Regarding the computational prediction method used in this paper, we made the method available to the public as a web-based service named COPICAT (COmprehensive Predictor of Interactions between Chemical compounds And Target proteins; http://copicat.dna.bio.keio.ac.jp/).

## Materials and Methods

### Experimental datasets

The DrugBank dataset was constructed from Approved DrugCards data, which were downloaded in February, 2007 from the DrugBank database [25]. These data consist of 964 approved drugs and their 456 associated target proteins, constituting 1,731 interacting pairs or positives.

### Computational prediction

#### Support vector machines

Given *n* samples, each of which has an *m*-dimensional feature vector (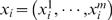) and one of two classes, such as binding and non-binding (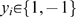), an SVM produces the classifier
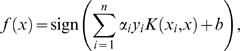
(2)where 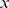 is any new object which needs to be classified, 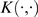 is a kernel function which indicates that the similarity between two vectors and (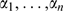) are the learned parameters [26]. The output of an SVM can be regarded as a probability [27].

#### First-layer SVM

In the first-layer SVM, a pair comprising a protein and a small molecule, which constitutes a sample, is mapped onto an *n*-dimensional numerical vector (feature vector) space by using amino acid sequences for proteins and 2D chemical structures for chemical compounds. Details regarding the numerical representation of the proteins and the chemical compounds are described in Text S1.

We generated 100 first-layer SVM models with different random combinations of proteins and chemical compounds as negatives. The SVM parameters were chosen to give the best accuracy in a 10-fold cross validation in one set of positives and negatives.

We prepared two sets of first-layer SVM models, each of which consists of 100 models. One set *allpos* contains the SVM models constructed from 1,731 positives, or the whole DrugBank dataset, and 1,750 negatives. The other set *subpos* is composed of models with 534 positives, one of 10 kinds of DrugBank subsets, and 550 negatives. A protein found *n* times in the DrugBank dataset is designed to appear ^▪^ *n*/10 ^▪^ +1 times in a DrugBank subset, and the chemical compounds with which the protein forms a pair differ between different subsets.

#### Second-layer SVM

The second-layer SVM directly utilizes the outputs of the first-layer SVM models as inputs. The second-layer SVM model was constructed from the whole DrugBank dataset and reasonably designed negatives, which are described in detail later, on the basis of the RBF kernel 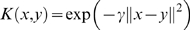 in Eq. (2). The SVM parameters were selected in such a way that they gave the best accuracy in the 10-fold cross validation. A schematic illustration of the second-layer SVM is shown in Figure S1.

#### Feature selection

The number of first-layer SVM models whose output is used in the second-layer SVM models mainly determines the computation time and the workload of the two-layer SVM methods. Therefore, in order to practically realize comprehensive protein-chemical interaction predictions, fewer first-layer models achieving high prediction accuracy are given preference.

We applied the recursive feature elimination (RFE) method [28] in order to determine the first-layer SVM models used to construct the second-layer SVM model. Details are shown in Text S1 and Figure S3.

#### Negative data design

We followed and modified the method described in Wang *et al.*, 2006 [29] for the design of negative data leading to the reduction of the number of false positives. First, negative seeds were selected on the basis of the distances between positive samples and unspecified samples, or between all combinations of proteins and chemical compounds in the dataset other than positives. Second, the negative samples were extended sequentially according to the four expansion rules *min*, *mlt*, *mle* and *max* by using the outputs of the prediction model constructed from positive samples and tentative negative samples as follows,

- min: Top *L* samples in the ascending order of *p_i_*, *i*∈*U*-*N*
- max: Top *L* samples in the descending order of *p_i_*, *i*∈*U*-*N*
- mle: Top *L* samples in the descending order of *p_i_*, *i*∈*U*-*N* s.t. *p_i_*≤0.5
- mlt: Top *L* samples in the descending order of *p_i_*, *i*∈*U*-*N* s.t. *p_i_*<0.5

where *N* was a set of tentative negative samples, *U* was a set of all the possible combination of combinations of proteins and chemical compounds in the dataset except positive samples, and *p_i_* was a probabilistic output of SVM. More details are provided in Text S1.

### Experimental verification

#### Materials

Unless otherwise specified, all solvents and reagents were obtained from commercial suppliers.

In the plasmid preparation, pTriAR, a construct in which Androgen receptor (AR) cDNA is subcloned into the pTriEX-3 Neo vector, was provided by Taiho Pharmaceutical.

In the in vitro binding assay, dihydrotestosterone (DHT), flutamide, nilutamide, spironolactone and cortexolone were purchased from Sigma. Testosterone and bicalutamide were purchased from Wako Pure Chemical Industries. ZINC 04369595, MDPI 944, MDPI 1011, NSC 6129, MDPI 10314, 3-epiuzarigenin, ZINC 04026296, methandriol, vitamin D3, ZINC 03849821, P712100 and fluanisone were purchased from Namiki Shoji.

#### Preparation of MBP-ARC (Maltose Binding Protein tagged Androgen Receptor C-termini)

The gene sequences corresponding to the ligand-binding domain (609th a.a.–919th a.a.) of androgen receptor C-termini (ARC) were subcloned into pMALc-2x vector digested with *Hind*III and *BamH*I, and the maltose binding protein-fusion androgen receptor C-termini (MBP-ARC) was expressed in *E. coli* DH5a, and purified on amylose resin (BioLabs). Details are provided in Text S1.

Here, it is reported that an in vitro binding assay with ARC produced almost the same results as that with whole-length AR [30].

#### The in vitro binding assay - hydroxyapatite method

50 µg/ml MBP-ARC, 2 nM [^3^H]-DHT, and the indicated amount of test compounds were incubated for three hours. Then, the radioactivity of [^3^H]-DHT bound to MBP-ARC was measured with a scintillation counter. Details are provided in Text S1.

The concentration of the test compound to [^3^H]-DHT in which the measured radioactivity corresponded to 50% of that measured without the test compounds was regarded as IC_50_ of the test compound.

### Feedback strategy

Given *N_p_* positive and *N_n_* negative samples in known data and *M_p_* positives and *M_n_* negatives in additional or feedback data, a straightforward strategy for the integration of additional data into statistical training, such as SVM, is to train a statistical model based on a dataset consisting of *N_p_*+*M_p_* positives and *N_n_*+*M_n_* negatives. When the two-layer SVM strategy is applied, another strategy of feedback and supplement involves the utilization of an additional model based on additional data. In this strategy, the second-layer SVM is trained on the basis of *N_p_*+*M_p_* positives and *N_n_*+*M_n_* negatives, and a sample *si* in the second layer is represented as follows, 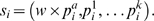
Here, 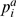 is an output of the additional model trained on the basis of *M_p_* positives and *M_n_* negatives. 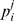 is an output of the first-layer SVM model *j*, and 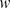 is a weighting factor.

### Docking analysis

AutoDock 4 [3] was applied to the human androgen receptor ligand-binding domain (PDB code; 2AM9 [31]) and tested compounds whose 3D structure was generated by Obgen in the Open Babel package ver.2.2.0 [32] or CORINA [33]. The conditions of AutoDock followed Jenwitheesuk and Samudrala, 2005 [34]. ARG752 of 2AM9, which was considered important for the binding of androgens by the human androgen receptor [31], was set to a flexible residue in AutoDock.

## Supporting Information

Text S1 Supplementary Methods and Supplementary Results are provided.
(0.13 MB PDF)

Figure S1 Schematic illustration of the two-layer SVM system.
(0.02 MB PDF)

Figure S2 Protein-drug interaction network for several datasets.
(0.38 MB PDF)

Figure S3 Effects of feature selection on two-layer SVM model.
(0.02 MB PDF)

Figure S4 Results of in vitro binding assay. Results of in vitro binding assay for each compound.
(0.62 MB PDF)

Figure S5 The scope of the third computational prediction.
(0.01 MB PDF)

Table S1 Prediction performances in several datasets
(0.03 MB PDF)

Table S2 Effects of integrating different types of protein-chemical interactions
(0.02 MB PDF)

Table S3 Prediction performances on different designed negatives
(0.02 MB PDF)

Table S4 Evaluation of our prediction method on an external test set
(0.06 MB PDF)

Table S5 Evaluation of our method with respect to comprehensive interaction prediction
(0.01 MB PDF)

Table S6 Utilization of one-class SVM in the selection of negative samples
(0.03 MB PDF)

Table S7 Overlaps of predictions between prediction models in Table S5
(0.03 MB PDF)
