# Supplementary material for: Integrating Statistical Predictions and Experimental Verifications for Enhancing Protein-Chemical Interaction Predictions in Virtual Screening
Source: PLoS Comput Biol. 2009 Jun 5;5(6):e1000397. doi: 10.1371/journal.pcbi.1000397 (PMC2685987; doi:10.1371/journal.pcbi.1000397)
Supplement: Text S1 — Supplementary Methods and Supplementary Results are provided. (0.13 MB PDF) [file pcbi.1000397.s001.pdf]

Table S5 Evaluation of our method with respect to comprehensive interaction prediction

[1]dataset	[2]neg.	[3]firsts	[4]P10275	[4]P11229	[4]P35367	[5] $\mathrm{rec}_{0.5}$(%)	[5] $\mathrm{rec}_{0.95}$(%)	[6]evaluation
(A) one-layer SVM								
mlt	16	–	714	1408	1187	100	98.97	82.50
mlt	14	–	709	1820	1634	100	*97.94	*79.02
max	16	–	4073	5956	6964	82.47	*56.70	*47.51
random	14	–	1896.7(±53.6)	10627.3(±648.9)	10204.0(±640.7)	100	99.66(±1.09)	69.20(±0.57)
random	16	–	1869.3(±136.1)	10503.3(±1250.7)	9305.3(±517.8)	100	99.66(±1.09)	69.45(±0.32)
(B) two-layer SVM-*subpos*								
mlt	14	10	177	535	451	96.91	93.81	75.56
mlt	14	11	205	671	491	96.91	91.75	73.54
mlt	14	9	239	513	403	95.88	91.75	73.87
mlt	14	8	290	456	363	88.66	82.47	66.58
mlt	12	10	224	561	612	95.88	92.78	73.25
mlt	16	10	162	466	415	94.85	89.69	73.47
min	14	10	2525	6098	3326	97.94	96.91	69.52
mle	14	10	168	526	599	97.94	92.78	74.79
max	14	10	32	386	191	92.78	*85.57	*72.27
random	14	10	848.3(±345.0)	1531.7(±628.9)	988.0(±411.4)	96.56(±2.89)	81.10(±19.44)	66.44(±7.82)
(C) two-layer SVM-*allpos*								
max	16	9	28	231	129	100	97.94	82.92
max	16	10	29	238	131	100	98.97	82.73
max	16	8	29	243	133	100	96.91	82.09
max	14	9	29	243	129	100	96.91	82.00
mle	16	9	28	267	140	100	100	80.99
mlt	16	9	67	248	141	100	100	80.72
random	16	9	74.7(±42.6)	255.3(±32.2)	146.7(±8.3)	100	100	80.67(±0.93)
(D) only compound SVM[7]								
–	–	–	640	1791	838	86.60	71.13	59.66
(E) similarity search[8]								
–	–	–	1869	1816	1580	–	–	–

[1] refers to negative data expansion rules (details are provided in Sec. 1.3 in Supplementary Materials). "random" indicates that three types of random pairs comprising a protein and a drug are used as negatives. The 95% confidence intervals are shown.
[2]: the number of negatives (= 1,750×x).
[3]: the number of the first-layer SVM models utilized for the construction of the second-layer SVM model.
[4]: target proteins whose ligands were predicted on the basis of 109,841 compounds. The number of predicted binding compounds is shown.
[5]: rec_x is the recall rate (=TP/(TP+FN)) at the threshold x, ranging from 0 to 1. 0.5 is the threshold following the definition of SVM. TP: true positives, FN: false negatives.
[6]:

$$\text{evaluation} = 100 \times \left(\frac{1}{2} \left[\mathrm{rec}_{0.5} + \frac{\mathrm{rec}_{0.95} + \mathrm{prec}_{0.95}}{2\{1 + (1 - \mathrm{rec}_{0.95})(1 - \mathrm{prec}_{0.95})\}} \right] - \frac{\text{total \# of predicted positives - \# of known positives}}{\text{total \# of prediction targets - \# of known positives}} \right)$$

Here, prec_x is the precision (=TP/(TP+FP)) at the threshold x. FP: false positives.
[7]: SVM model in which chemical compounds binding to each target protein were treated as positives and all other compounds in the DrugBank dataset were regarded as negatives.
[8]: A chemical compound i was predicted as a binding ligand of a protein α by using the similarity method if $pred_{\mathrm{sim}}(i) = \max_{j \in A} |I \cap J|/|I \cup J| \geq 0.9$, where A represents the known binding ligands of the protein α, and I (or J) represents a set of substructures considered in calculating the feature vector of the chemical compounds.
*: the threshold was set to 0.9 instead of 0.95 for the calculation of "evaluation".
