# Supplementary material for: Integrating Statistical Predictions and Experimental Verifications for Enhancing Protein-Chemical Interaction Predictions in Virtual Screening
Source: PLoS Comput Biol. 2009 Jun 5;5(6):e1000397. doi: 10.1371/journal.pcbi.1000397 (PMC2685987; doi:10.1371/journal.pcbi.1000397)
Supplement: Figure S1 — Schematic illustration of the two-layer SVM system. (0.02 MB PDF) [file pcbi.1000397.s002.pdf]

Contents

1 Supplementary Methods 2
1.1 Experimental datasets . . . 2
1.2 Two-layer SVM . . . 2
1.3 Negative data design . . . 4
1.4 Strategy of feedback and supplement with additional data . . . 4
1.5 Target protein - androgen receptor . . . 5
1.6 Materials . . . 5
1.7 Plasmid preparation . . . 5
1.8 Recombinant ARC protein preparation and purification . . . 6
1.9 The *in vitro* binding assay - hydroxyapatite method . . . 6

2 Supplementary Results 7
2.1 Supplementary indication of biological validity of statistical approaches . . . 7
2.2 Construction of two-layer SVM model . . . 7
2.3 Construction of designed negative data . . . 7
2.4 Evaluation of our proposed prediction method . . . 8
2.5 Supplementary false positive reduction in comprehensive prediction . . . 8
2.6 Comparison with the negative data design on the basis of one-class SVM . . . 9
2.7 Comparison with other prediction approaches . . . 10
2.8 Overlaps of predictions between prediction models . . . 10
2.9 Supplementary application of our strategy to the discovery of androgen receptor binding ligands . . . 10

Abbreviations

AR: androgen receptor, DHT: dihydrotestosterone, ARC: androgen receptor C-termini, MBP-ARC: maltose binding protein tagged androgen receptor C-termini, SVM: support vector machine, ANN: artificial neural network, QDA: quadratic discriminant analysis

1 Supplementary Methods

Computational experiment section

1.1 Experimental datasets

The DrugBank dataset was constructed from Approved DrugCards data, which was downloaded in February, 2007, from the DrugBank database (Wishart *et al.*, 2008). These data consist of 964 approved drugs and their 456 associated target proteins, constituting 1,731 interacting pairs or positives.

1.2 Two-layer SVM

1.2.1 Support vector machines

Given n samples, each of which has a m-dimensional feature vector ($\boldsymbol{x}_i = (x_i^1, \ldots, x_i^m)$) and one of two classes such as binding and non-binding ($y_i \in \{1, -1\}$), an SVM produces the classifier

$$f(\boldsymbol{x}) = \text{sign}\left(\sum_{i=1}^{n} \alpha_i y_i K(\boldsymbol{x}_i, \boldsymbol{x}) + b^*\right), \tag{1}$$

where $\boldsymbol{x}$ is any new object which needs to be classified, $K(\cdot, \cdot)$ is a kernel function which indicates the similarity between two vectors and $(\alpha_1, \ldots, \alpha_n)$ are the learned parameters (Vapnik, 1998).

The output of an SVM can be regarded as a probability using the following formula (Platt, 2000),

$$p(y = 1|\boldsymbol{x}) = \frac{1}{1 + \exp\left(A(\sum_{i=1}^{n} \alpha_i y_i K(\boldsymbol{x}_i, \boldsymbol{x}) + b^*) + B\right)} \tag{2}$$

A and B are parameters given by solving the likelihood maximization.

In this report, the *LIBSVM* 2.81 (Chang and Lin, 2001) program was employed to construct the SVM model.

1.2.2 First-layer SVM

In order to apply the support vector machine expressed in Eq. (1) to non-numerical data including amino acid sequences and chemical structures, this type of data must be converted into some numerical data. In the first-layer SVM, a pair comprising a protein and a small molecule, which constitutes a sample, is mapped onto n-dimensional numerical vector (feature vector) space by using amino acid sequences for proteins and 2D chemical structures for chemical compounds.

A feature vector for a protein p, $C(p)$, is calculated as follows,

$$C(p) = (\rho_p(c))_{c \in \mathcal{C}}, \quad \rho_p(c) = \begin{cases} \dfrac{f_p(c)}{\sum\limits_{i \in \mathcal{C}(p)} f_p(i)} & \text{if } c \in \mathcal{C}(p) \\ 0 & \text{otherwise} \end{cases} \tag{3}$$

where c is one of 199 clusters in which amino acid timers with similar physio-chemical properties are clustered. $\mathcal{C}$ is a set of clusters that appear at least once in proteins in the dataset and $\mathcal{C}(p)$ is a set of clusters observed at least once in a protein p. $f_p(c)$ is the number of appearances of a cluster c in a protein p. (More details are in Ref. (Nagamine and Sakakibara, 2007))

For example, the five-letter amino acid sequence $NGMGN$ can be represented as follows,

$$C(NGMGN) = \begin{pmatrix} c(A(AA)) & & c(G(MN)) & & c(M(GG)) & & c(Y(YY)) \\ 0, & \ldots, & 2/3, & \ldots, & 1/3, & ,\ldots, & 0 \end{pmatrix}.$$

where $c(s)$ is a cluster to which a signature s belongs.

A feature vector for a chemical compound c is defined as follows,

$$D_l^h(c) = (\psi_c(p))_{p \in \mathcal{P}_l^h}, \quad \psi_c(p) = \begin{cases} \dfrac{f_c(p)}{\sum\limits_{i \in \mathcal{P}_0^h(c)} f_c(i)} & \text{if } \boldsymbol{p} \in \mathcal{P}_l^h(c) \\ 0 & \text{otherwise} \end{cases} \tag{4}$$

where p is a "path", or a substructure extracted from chemical structures, which are regarded as a graph with an atom as a node and a bond as an edge. $\mathcal{P}_l^h$ is a set of paths whose depth, or a number of bonds within, is between l and h ($h \geq l$) and which appears at least once in chemical structures in the dataset and $\mathcal{L}_l^h(c)$ is that found at least once in a chemical c. $f_c(p)$ is a number of appearances of path p in the structure of chemical compound c. In relation with C, 199 most fluctuating paths were selected from $\mathcal{P}_l^h$ to encode chemical compounds. (More details are in Ref. (Nagamine and Sakakibara, 2007))

For example, methane (CH_4) can be represented as follows,

$$\mathrm{CH_4} = \begin{array}{c} \mathrm{H} \\ | \\ \mathrm{H{-}C{-}H} \\ | \\ \mathrm{H} \end{array}, \quad D_0^2(\mathrm{CH_4}) = \begin{array}{c} \\ (\end{array}\!\!\begin{array}{cccccccccc} \mathrm{(C)} & \mathrm{(H)} & \mathrm{(O)} & \mathrm{(CsH)} & & \mathrm{(OsH)} & & \mathrm{(HsCsH)} & & \mathrm{(OdCsO)} \\ 1/15, & 4/15, & 0, & 4/15, & \ldots, & 0, & \ldots, & 6/15, & \ldots, & 0 \end{array}\!\!\begin{array}{c} \\ ). \end{array}$$

For two samples $\boldsymbol{x}$ and $\boldsymbol{x}'$, or pairs of a protein and a compound, expressed as $\boldsymbol{x} = (C, D)$ and $\boldsymbol{y} = (C', D')$, the similarity between them or $K(\boldsymbol{x}, \boldsymbol{x}')$ in Eq. (1) is defined to consider combination effects as follows (More details are in Ref. (Nagamine and Sakakibara, 2007)),

$$K\{(C, D), (C', D')\} = \prod_{I \in \{C,D\}, J \in \{C',D'\}} k_{IJ=JI}(I, J), \quad k_{ij}(\boldsymbol{x}, \boldsymbol{y}) = \exp(-\gamma_{ij} \|\boldsymbol{x} - \boldsymbol{y}\|^2). \tag{5}$$

Based on feature vectors for samples expressed in Eqs. (3) and (4) and similarity between two samples defined in Eq. (5), we generated 100 first-layer SVM models with different random combination of proteins and chemical compounds as negatives. The SVM parameters were chosen to give the best accuracy in 10-fold cross validation in one set of positives and negatives.

We prepared two sets of first-layer SVM models, each of which consists of 100 models. One set *allpos* contains the SVM models constructed from 1,731 positives, or the whole DrugBank dataset (*allpos* first-layer SVM models where D_0^l in Eq. (4) was used), and 1,750 negatives. The other set *subpos* is composed of models with 534 positives, one of 10 kinds of DrugBank subsets, and 550 negatives (*subpos* first-layer SVM models where D_2^l in Eq. (4) was used). A protein found n times in the DrugBank dataset is designed to appear $\lfloor n/10 \rfloor + 1$ times in a DrugBank subset, and the chemical compounds with which the protein forms a pair differ between different subsets.

1.2.3 Second-layer SVM

The second-layer SVM directly utilizes the outputs of the first-layer SVM models as inputs. The second-layer SVM model was constructed from the whole DrugBank dataset and reasonably designed negatives, which are described in detail later in Sec. 1.3 on the basis of the RBF kernel $K(\boldsymbol{x}, \boldsymbol{y}) = \exp(-\gamma \|\boldsymbol{x} - \boldsymbol{y}\|^2)$ in the Eq. (1). The SVM parameters were selected in such a way that they gave the best accuracy in the 10-fold cross validation. Finally, the two-layer SVM functions as shown in Fig. S1.

For comparison of classification methods, quadratic discriminant analysis (QDA) and artificial neural network (ANN), which were implemented by R functions *qda* and *nnet* (Venables and Ripley, 2002) (`http://cran.r-project.org/`), were applied to outputs of the first-layer SVM models.

1.2.4 Feature selection

The number of the first-layer SVM models whose output is used in the second-layer SVM models mainly determines the computation time and the workload of the two-layer SVM methods. Therefore, in order to practically realize comprehensive protein-chemical interaction predictions, fewer first-layer models achieving the high prediction accuracy are given preference.

We applied the recursive feature elimination (RFE) method (Xue *et al.*, 2004) in order to determine the first-layer SVM models used to construct the second-layer SVM model. When n (=100 for the first time) first-layer models are considered, the model i satisfying the following criterion is eliminated to produce the second-layer SVM model with $n - 1$ dimensions.

$$\operatorname*{argmin}_i \ \frac{1}{2}\boldsymbol{\alpha}^t H(0)\boldsymbol{\alpha} - \frac{1}{2}\boldsymbol{\alpha}^t H(i)\boldsymbol{\alpha}, \ H(i) = \begin{bmatrix} K'_{11} & \ldots & K'_{1n} \\ \vdots & \ddots & \vdots \\ K'_{n1} & \ldots & K'_{nn} \end{bmatrix}, \ K'_{jk} = y_j y_k \exp\left(-\gamma \sum_{l=1, l \neq i}^{m} (x_{jl} - x_{kl})^2\right), \tag{6}$$

where $\boldsymbol{\alpha}$ is the same as those in Eq. (1). This elimination continues until n reaches a certain number.

1.3 Negative data design

We followed and modified the method described in Ref. (Wang *et al.*, 2006) for the design of negative data leading to the reduction of the number of false positives.

From P:(positive samples or the DrugBank dataset), U:(all the possible combinations of proteins and chemical compounds found in the DrugBank dataset except positive samples), $N = \emptyset$, we constructed the designed negative dataset N through following two phases.

(Phase A) Determination of negative dataset seed.

1. Add a sample i satisfying the following criterion to N.

$$\underset{i}{\text{argmax}}\, d(\boldsymbol{x}_i, P), \quad d(\boldsymbol{x}_i, P) = \min_{\boldsymbol{x}_j \in P} \|\boldsymbol{x}_i, \boldsymbol{x}_j\|$$

2. Add a sample i satisfying the following criterion to N.

$$\max_{\boldsymbol{x}_i \in (U-N)} \left[d(\boldsymbol{x}_i, P) \cdot \sum_{\boldsymbol{x}_j \in N} d(\boldsymbol{x}_i, \boldsymbol{x}_j) \right], \quad d(\boldsymbol{x}_i, \boldsymbol{x}_j) = \|\boldsymbol{x}_i, \boldsymbol{x}_j\|$$

3. Repeat the step 2 until $|N|$ reaches a certain number ($|N| = 3500 \approx 2 \times |P|$).

(Phase B) Expansion of negative dataset.

1. Construct a SVM model from P and N.
2. The constructed SVM model is applied to $U - N$. L samples ($|L| = 3500 \approx 2 \times |P|$) are added to N according to the probabilistic output p_i of SVM expressed in Eq. (2) and following rules.
   min: Top L samples in the ascending order of p_i, $i \in U - N$.
   max: Top L samples in the descending order of p_i, $i \in U - N$.
   mle: Top L samples in the descending order of p_i, $i \in U - N$ s.t. $p_i \leq 0.5$.
   mlt: Top L samples in the descending order of p_i, $i \in U - N$ s.t. $;p_i < 0.5$.
3. Repeat the step 1 and 2.

Particularly, the rules *max*, *mle* and *mlt* were introduced for significant false positive reduction.

1.4 Strategy of feedback and supplement with additional data

Computational predictions by statistical methods , docking methods or molecular dynamics methods involve success and failure after they are verified by biological experiments. One of the merits of using statistical methods involving training with known data is that results obtained by verification experiments can be efficiently utilized or feedbacked to produce newer and more reliable predictions. Moreover, without verification experiments, additional data can be acquired from, for example, literature and used to enhance prediction reliability.

Given N_p positive and N_n negative samples in known data and M_p positives and M_n negatives in additional or feedback data, a straightforward strategy for the integration of additional data in statistical training such as SVM is to train a statistical model based on a dataset consisting of $N_p + M_p$ positives and $N_n + M_n$ negatives. When the two-layer SVM strategy is considered, another strategy of feedback and supplement involves the utilization of an additional model based on additional data. In this strategy, the second-layer SVM is trained on the basis of $N_p + M_p$ positives and $N_n + M_n$ negatives, and a sample s_i in the second-layer is represented as follows,

$$s_i = (w \times p_{ia}, p_{i1}, \ldots, p_{ik}).$$

Here, p_{ia} is an output of the additional model trained on the basis of M_p positives and M_n negatives. p_{ij} is an output of the first-layer SVM model j and w is a weighting factor.

For three proteins; UniProt ID P10275 (androgen receptor), P11299 (muscarinic acetylcholine receptor M1) and P353367 (histamine H1 receptor), ligand data that was not included in the DrugBank dataset were collected from literature (Funder and Mercer, 1979; Link *et al.*, 2005; Kinoyama *et al.*, 2006) and public databases; PDSP Ki database (Roth *et al.*, 2000) and GLIDA (Okuno *et al.*, 2008) in February 2008. Overall, 35 androgen receptor-ligand pairs, 49 muscarinic acetylcholine receptor M1-ligand pairs

and 1,060 histamine H1 receptor-ligand pairs were supplemented. Additional models were constructed using these supplemental pairs as positives and regarding pairs of each protein and chemical compounds in the DrugBank dataset other than binding ligands and those in negative dataset seed described in Sec. 1.3, or pairs least likely to interact, as negatives. Roughly the same number of these two types of negatives were utilized. When these supplemental pairs had been regarded as negatives in the process of selection of candidates for negatives, these samples were treated as positives.

Biological experiment section

1.5 Target protein - androgen receptor

Androgen receptor (AR) is one of genes responsible for prostate cancer, which is the most frequently diagnosed cancer in men in the United States according to the American Cancer Society Statistics for 2008. AR is a steroid hormone receptor and a transcription factor belonging to the nuclear receptor superfamily. AR protein consists of the N-terminal domain that contains the activation function 1 region and regulates the transcription activity (Danielian *et al.*, 1992), the DNA binding domain at the central, the ligand binding domain at the C-termini, and the hinge region containing nuclear localization signals between these binding domains. In prostate cancer, mutations in the AR gene, overexpression of the AR proteins, and further suppression of cancer cell proliferation by knockdown of the AR gene induced by siRNA were observed (Compagno *et al.*, 2007).

In prostate cancer therapy, hormone therapy using an androgen antagonist such as flutamide, nilutamide and bicalutamide exists. These drugs are indicated to cause severe side effects such as interstitial pneumonia and including liver disorders as AR is expressed in several tissues including the lungs and liver. However, a selective androgen receptor modulator, which acts as an antagonist in specific tissues and as an agonist in other tissues or vice versa, is expected to overcome side effects (Gao *et al.*, 2005).

As a mechanism of action of selective androgen receptor modulators is not fully elucidated and most of them have been found by chance (Chen *et al.*, 2002), it is necessary to efficiently identify a lot of compounds targeting AR and select potent antagonists of prostate cancer cells from them for the discovery of selective androgen receptor modulator drugs.

1.6 Materials

Unless otherwise specified, all solvents and reagents were obtained from commercial suppliers.

In the plasmid preparation, pTriAR, a construct in which Androgen receptor (AR) cDNA is subcloned into the pTriEX-3 Neo vector, was provided by Taiho Pharmaceutical Co., Ltd.

In the *in vitro* binding assay, dihydrotestosterone (DHT), flutamide, nilutamide, spironolactone and cortexolone were purchased from Sigma. Testosterone and bicalutamide were purchased from Wako Pure Chemical Industries, Ltd. ZINC 04369595, MDPI 944, MDPI 1011, NSC 6129, MDPI 10314, 3-Epiuzarigenin, ZINC 04026296, Methandriol, Vitamin D3, ZINC 03849821, P712100 and fluanisone were purchased from Namiki Shoji Co., Ltd.

1.7 Plasmid preparation

The gene sequences corresponding to the ligand-binding domain (609th a.a. - 919th a.a.) of androgen receptor C termini (ARC) were amplified by PCR with pTriAR as a template, 5′-ATGACTCTGGGAGCCCGG-3′ (sense) and 5′-CCCTCGAGTCACTGGGTGTGGAAATAGATGGG-3′ (anti-sense) primers, and KOD plus (Toyobo Co., Ltd.) as DNA polymerase. The PCR conditions were as follows: 40 cycles of denaturation (98 ℃, 15 seconds), annealing (60 ℃, 30 seconds) and extension (68 ℃, 3 minutes).

After agarose gel electrophoresis of PCR products, DNA fragments of supposed ARC were subcloned into pBlueScript II SK(-) vector (Stratagene) with *EcoRV*. This recombinant plasmid (pBS/ARC) was sequenced to verify that ARC was properly amplified.

After verification, ARC sequences were digested from pBS/ARC, and subcloned into pMALc-2x vector

digested with *HindIII* and *BamHI* to obtain a recombinant plasmid pMAL/ARC which expressed, in *E. coli*, the maltose binding protein tagged androgen receptor C-termini (MBP-ARC).

Here, it is reported that an *in vitro* binding assay with ARC produced almost the same result as that with the whole length AR (Zhu *et al.*, 2001).

1.8 Recombinant ARC protein preparation and purification

The pMAL/ARC plasmid was transfected into *E. coli* DH5α. The transfected cells were cultivated in LB medium containing 50 μg/ml ampicillin at 37 ℃ overnight. The culture solution was diluted by LB medium so that OD_{600} was equal to 0.1. After one hour cultivation of the diluted culture solution at 25 ℃, 0.1 mM IPTG was added to the solution. Then, the solution was cultivated at 25 ℃ for 12 hours.

After cultivation, the cells were harvested with centrifuge at 3,500 rpm for 30 minutes. The cells were then suspended in 30-ml MT-PBS containing 1 % Triton and disintegrated with an ultrasonic homogenizer.

After 20-minute centrifugation of the homogenized solution at 3,500 rpm, a supernatant was collected. The supernatant was further centrifuged at 13,000 rpm for 30 minutes to obtain a soluble fraction. The soluble fraction was applied to a 10-ml column of amylose resin (New England BioLabs), which was washed with a 50-ml column buffer consisting of 20 mM Tris-Hcol pH 7.4, 200 mM NaCl and 1mM EDTA. After column washing with the 125-ml column buffer containing 0.03 mM maltose, the recombinant ARC protein was eluted with 15-ml column buffer containing 10mM maltose. All the purification processes were carried out at 4 ℃.

1.9 The *in vitro* binding assay - hydroxyapatite method

50 μg/ml recombinant ARC protein , 2 nM [^{3}H]-DHT and a test compound in a molar ratio x:1 ($x = 0.01 - 3 \times 10^5$) with [^{3}H]-DHT were mixed in a binding buffer consisting of 50mM Tris-HCl pH 7.6, 800 mM NaCl, 10 % glycerol, 1mg/ml BSA and 2mM DTT to obtain a 100 μl mixture solution. The mixture solution was incubated at 4 ℃for 3 hours.

After incubation, BioGel HT (Bio-Rad Laboratories) was added to the solution and incubated on ice for 15 minutes, during which vortexing was done every 5 minutes. The incubated solution was centrifuged at 1,000 rpm for a minute and a supernatant was removed.

A wash buffer consisting of 40 mM Tris-HCl pH 7.6, 100 mM KCl, 1mM EDTA and 1mM EGTA and cooled on ice was added to the precipitate by 1 ml and mixed. The produced solution was centrifuged at 1,000 rpm and a supernatant was removed. This process was repeated three times.

The collected precipitate was suspended in 3 ml Aquasol (New England nuclear Corp.). Radioactivity of the suspended solution in a scintillation vial (Perkin Elmer) was measured by by a liquid scintillation counter.

Here, the radioactivity derived from [^{3}H]-DHT showed the amount of the recombinant ARC protein bound by [^{3}H]-DHT. As [^{3}H]-DHT and test compounds competitively were thought to bind to the recombinant ARC protein, decrease of radioactivity from that measured without competitors, or when [^{3}H]-DHT were thought to bind to all the recombinant ARC protein, reflected the content of the recombinant ARC protein bound by unlabeled competitors. Therefore, the concentration of the test compound to [^{3}H]-DHT in which the measured radioactivity corresponded to 50 % of that measured without the test compounds was regarded as IC_{50} of the test compound.

2 Supplementary Results

2.1 Supplementary indication of biological validity of statistical approaches

In several datasets consisting of known pairs of proteins, including nuclear receptors, GPCRs, ion channels and enzymes, and drugs as positives samples and random protein-drug pairs as negatives, our statistical approach with SVM showed high prediction performances (Table S1). The fact that more than 0.85 AUC and an accuracy of 80% were obtained for diverse datasets suggests that it is possible to extract some properties accountable for interactions between proteins and drugs by statistical approaches.

The possibility that statistical approaches can derive some fundamental protein-chemical binding rules can be further supported by the fact that integrating several datasets described in Table S1 improved the prediction performances on a test set, which was a part of one dataset (Table S2). If statistical methods can only extract binding rules specific to a protein or, at most, to a protein family, the prediction model on the basis of pairs of drugs and proteins belonging to several different protein families will achieve prediction performances as good as or lower than the model based on pairs of drugs and proteins belonging to a protein family when applied to prediction targets consisting of proteins belonging to the protein family and their drugs. In Table S2, use of GPCR-drug pairs and ion channel-drug pairs in addition to nuclear receptor-drug pairs improved the prediction performances on the test sets consisting of nuclear receptor-drug pairs. These results support general applicability of statistical approaches in protein-chemical interactions and encourage the use of datasets comprising several types of protein families to construct statistical prediction models.

2.2 Construction of two-layer SVM model

We generated twelve two-layer SVM models (three types of different random pairs as negatives each for four negative sample numbers; 3,500, 7,000, 10,500 and 14,000) to verify the effects of feature selection (Fig. S3). As shown in Fig. S3A, reduction of *subpos* first-layer SVM models generally elevates and maintains high prediction accuracy. As far as second-layer SVM models utilized more than 10 first-layer SVM models, changes of prediction accuracy were limited to $\pm 0.2\%$.

These first-layer SVM models were evaluated based on results of feature selection by the following equation (Fig. S3B).

$$\text{model\_evaluation}(i) = \sum_{c=1}^{12} \sum_{j=1}^{100} (acc_{cj}/j) \times s_j(i), \quad s_j(i) = \begin{cases} 1 & \text{if } i \in M_{cj} \\ 0 & \text{otherwise,} \end{cases} \tag{7}$$

where acc_{cj} is the accuracy obtained by applying j first-layer SVM models to the dataset c, and M_{cj} is a set of these j models. Fig. S3B shows that contribution to classification markedly differs among the models despite a variety of negative data. This suggests that there exist some combination of first-layer SVM models applicable to a wide range of datasets with no or small loss of prediction performances.

2.3 Construction of designed negative data

Based on the preceding findings, we selected 35 first-layer SVM models, which gave the largest accuracy elevation in Fig. S3A, according to the descending order of the model evaluation in Fig. S3B. These 35 first-layer SVM models were applied to all the possible combinations of proteins and drugs in the DrugBank dataset. The method described in Sec. 1.3 was applied to the yielded $\mathcal{X} = \{\boldsymbol{x}_1 = (p_{1,1}, \ldots, p_{1,35}), \ldots, \boldsymbol{x}_{439584}\}$.

Table S3 shows prediction performances on datasets produced according to four different rules described in Sec. 1.3. As shown in Table S3, the dataset *min* gave the best accuracy and the dataset *max* did the worst. As the expanding rule *max* in Sec. 1.3 is designed to construct the dataset which is the most difficult to be classified and the rule *min* is intended for the easiest dataset, these results are reasonable enough.

2.4 Evaluation of our proposed prediction method

Though Table S3 exhibits very high performances, these results are possibly overestimated as the same positives and negatives were allowed in datasets used to construct first-layer SVM models and second-layer SVM models. Therefore, we tested our prediction method on an external dataset (Table S4).

In Table S4, the external dataset consisted of 170 positives and 2,450 negatives that were randomly chosen from 1,731 positives and 24,500 designed negatives with the *mlt* rule and that were excluded in constructing first-layer and second-layer SVM models. Here, "one-layer" SVM model was produced based on the same features as used for the first-layer SVM model. To construct the second-layer SVM model, 11 *subpos* or *allpos* first-layer SVM models, which were chosen by the feature selection method described in Sec. 1.2, were utilized.

We evaluate prediction performances with default or higher threshold as prediction results of comprehensive application are often processed with such thresholds. Among several evaluation measures, precision, or reliability of positive prediction, is relevant for measuring the effects of false positive reduction.

As shown in Table S4A, designed negatives contribute to better prediction performances. Use of rationally designed negatives instead of randomly chosen negatives significantly improved precision of external prediction by more than 20%. Comparison with *subpos* and $subpos_r$ in Table S4B also exhibited improvement of precision by introducing designed negatives.

Table S4B shows effectiveness of utilizing the second-layer SVM model. Compared with simplest ways to integrate outputs of several first-layer SVM models including voting ($subpos_{\mathrm{v}}$) and averaging ($subpos_{\mathrm{m}}$), the use of second-layer SVM (*subpos*) model gave highly better overall prediction performances. Besides, though the use of higher threshold leads to better precision at the risk of sensitivity, $subpos_{\mathrm{v}_{0.8}}$ and $subpos_{\mathrm{m}_{0.8}}$ still exhibited lower precision than the second-layer SVM model. Here, the higher threshold (e.g. $subpos_{\mathrm{v}_{0.9}}$) produced no positives.

Other statistical classification methods can be applied to outputs of the first-layer SVM models. In comparison to other major non-linear classification methods including artificial ($subpos_{\mathrm{ann}}$ in Table S4B) and quadratic discriminant analysis ($subpos_{\mathrm{qda}}$) (Venables and Ripley, 2002), the use of SVM (*subpos*) exhibits better performances in external prediction. These findings show the effectiveness of utilizing SVM to process outputs of the first-layer SVM models.

As described in Sec. 1.2, we conducted feature selection in constructing the second-layer SVM model. The observation that the second-layer SVM model with feature selection (*subpos* in Table S4B) exhibited, in external prediction, significantly higher precision (P-value = 0.0081 by t test) and sensitivity (P-value $= 1.8 \times 10^{-9}$ by t test) than that with randomly selected first-layer SVM models supports utilization of the feature selection.

Table S4C exhibits that the second-layer SVM approach improved precision and suggests that use of *allpos* first-layer SVM models lead to significant reduction of false positives. The second-layer SVM model with *allpos* first-layer SVM models (*allpos* in Table S4C) achieved 100% precision though prediction sensitivity was low. In general, higher precision can be achieved at the risk of sensitivity by using higher threshold. However, even with higher threshold (e.g. $t = 0.9$), 100% precision weren't realized by the one-layer SVM and the second-layer SVM with *subpos* first-layer SVM models (one-layer$_{0.9}$ and $subpos_{0.9}$). Therefore, the use of first-layer SVM models, particularly *allpos* models, in the two-layer SVM approach is considered to contribute to meaningful improvement of precision and reduction of false positives.

2.5 Supplementary false positive reduction in comprehensive prediction

It is often observed that although statistical learning approaches achieve very high prediction performances in given datasets, statistical prediction models suffer from the problem of generating vast prediction sets including many false positives when applied to comprehensive prediction. In our approach, SVM models based on feature vectors directly representing amino acid sequences, chemical structures, and random protein-compound pairs as negatives also produced many predictions and inevitably yielded many false positives (Table S5A *random*).

Upon the introduction of the two-layer SVM and the negatives designed to overcome this drawback, the prediction precision, or the confidence of positive prediction, was significantly improved in computational experiments based on the DrugBank dataset (details are provided in Supplementary Material). The high precision contributes to the selection of more reliable predictions and thus to the reduction of the number of false positives.

Following these results on given datasets, our approaches were evaluated with respect to comprehensive binding ligand prediction. For three proteins (P10275 (androgen receptor), P11299 (muscarinic acetylcholine receptor M1) and P35367 (histamine H1 receptor), their binding ligands were predicted from PubChem Compound 0000001–00125000 which contains 109,841 compounds (Table S5). Here, P35367 and P11299 are the two most frequently targeted proteins in the DrugBank dataset, and P10275 is a protein of average appearance in the DrugBank dataset. Among the 109,841 compounds, 47, 45, and 5 known ligands were included for P35367, P11299, and P10275, respectively.

In Table S5, we propose a measure ("evaluation") to evaluate models. This measure is based on the following three ideas. First, only prediction results beyond some high threshold are frequently considered. Secondly, the prediction of 20% sensitivity and 80% precision is often preferred to that of 50% sensitivity and 50% precision. Thirdly, prediction with not too many candidates and high sensitivity at some lower threshold can be a target for comprehensively applicable experimental methods.

As shown in Table S5A, utilization of designed negative data (e.g. *mlt* dataset) decreased a number of predicted compounds with a slight loss of sensitivity. Therefore, it is considered to contribute to significant reduction of false positives while detecting the majority of true positives.

In comparison of results for *random* datasets in Table S5A, S5B and S5C , introduction of the two-layer SVM method also shows effects on reduction of false positives. The utilization of *allpos* first-layer SVM models (Table S5C) decreased a number of predicted compounds more significantly without loss of sensitivity than that of *subpos* first-layer SVM models, which showed a small loss of sensitivity.

Furthermore, as shown in Table S5A, S5B and S5C, integration of these two effective approaches, i.e. utilization of rationally designed negatives and introduction of the two-layer SVM, reduces false positives more efficiently. For example, in comparison to Table S5A and S5C, the number of candidates discovered by using the *max* dataset in the *allpos* two-layer SVM approach was about one fiftieth of the number of chemical compounds predicted by using the *random* dataset in one-layer SVM.

The degree of reduction, sensitivity and precision depend on datasets. For example, use of the dataset *min* gave more predicted compounds than random datasets as it was constructed from positives and negatives that were distant from each other (Table S5B). On the other hand, utilization of the datasets *mlt*, *mle* or *max* reduced candidate compounds. These datasets were constructed by taking in samples that were difficult to classify by existing SVM models as negatives, and expected to reduce false positives by forming stringent classification boundaries. This result suggests that such concept for negative data design contribute to false positive reduction in comprehensive application of classification methods.

About a number of the first-layer SVM models and negatives, around 10 and around mid-tens-fold of positives respectively were appropriate in this experiment. These values may differ among applications.

According to our proposed measure, one-layer SVM using the *mlt* dataset with 28,000 negatives, two-layer SVM using 10 *subpos* first-layer SVM models and the *mlt* dataset with 24,500 negatives and two-layer SVM using 9 *allpos* first-layer SVM models and the *max* dataset with 28,000 negatives can be candidates for the comprehensively applicable protein-chemical interaction prediction model.

Moreover, in comparison to other approaches based only on the properties of chemical compounds (Tables S5D and S5E), our approaches gave a reasonable number of predictions.

These results suggest that our prediction models select a reasonable number of ligand candidates from all chemical compounds in large databases, and encourage the comprehensive binding ligand prediction for the target protein.

2.6 Comparison with the negative data design on the basis of one-class SVM

One-class SVM (Scholkopf *et al.*, 2001) estimates high-density regions from data samples and is used to detect outliers. A one-class SVM model trained on positive samples can be applied to unspecified samples in order to estimate plausible negatives by selecting samples from outliers. On the other hand,

choosing negatives from unspecified samples near the high-density region can contribute to formation of stringent classification boundaries.

In comparison of Table S6B and S5B, our method of selecting candidates for negative samples, which involved a phased increase of negative samples on the basis of generated classification boundaries, outperformed that of using the one-class SVM.

2.7 Comparison with other prediction approaches

Most previous methods are based on only information of chemical compounds and similarity searches. While our proposed SVM model can deal with any proteins by itself, it is necessary to construct an SVM model for each protein with known binding ligands as positives.

In Table S5D, "only compound SVM" models for each protein were produced by using D_2^6 in Eq. (4) to represent chemical compounds. Here, the same mapping was used for "only compound" and first-layer SVM models to evaluate classification setting itself. It was based on datasets constituted with known approved drugs for each protein as positives and other chemical compounds found in the dataset of binding pairs as negatives. As shown in Table S5D, these "only compound SVM" models exhibited worse prediction performances than our proposed methods. These findings show that consideration of a pair of a protein and a chemical compound can be useful in predicting binding ligands for proteins.

Moreover, Table S5E exhibits predictions made by a similarity search. Here, a chemical compound i is predicted as a binding ligand of a protein α if

$$pred_{\mathrm{sim}}(i) = \max_{j \in A} \frac{|I \cap J|}{|I \cup J|} \geq 0.9,$$

where A is known binding ligands of the protein α, and I (or J) is a set of substructures in $\mathcal{P}_2^6(i)$ (or $\mathcal{P}_2^6(j)$) described in Eq. (4). Compared with Table S5E, our proposed methods (e.g. the two-layer SVM in Table S5B, C) gave a smaller number of candidates, which included chemical compounds that weren't found by the similarity search (Table S7). These results suggest that, in terms of selection of, preferably novel, candidates to be experimentally verified and reduction of false positives, our proposed methods can achieve these purposes more efficiently than mere similarity searches.

2.8 Overlaps of predictions between prediction models

Table S7 exhibits overlaps of predicted chemical compounds between prediction models. Our proposed methods shared more than half of their predictions with the only compound SVM. Therefore, consideration of pairs is relevant to utilization of only chemical compound information, or well used ligand based virtual screening approach. With differences of candidates that they cover, they can complement each other.

Our methods tried to reduce false positives, and thus, as shown in Table S7, predicted a smaller number of candidates than mere similarity search, which is based on the assumption that similar compounds have similar functions. The observation that more than half of candidates predicted by our method were structurally similar to known binding ligands and that the ratio increased with higher threshold partly shows validity of our prediction models, and effects of our methods on efficient candidate selection. On the other hand, the fact that even the least productive or the most stringent model (*allpos*) gave candidates that weren't similar to known ligands implies possible contribution of our methods to discovery of completely novel ligands.

2.9 Supplementary application of our strategy to the discovery of androgen receptor binding ligands

2.9.1 Supplementary results of the first and second experimental verification

For 23 compounds tested in the first and second experimental verification, chemical structures and results of *in vitro* binding assay were shown in Fig. S4.

2.9.2 Third computational prediction

The results of the second experimental verifications were feedbacked to re-construct the third prediction model. In constructing this prediction model, some compounds that had structures similar to steroid skeletons were also regarded as negatives. As shown in Fig. S5, this model produced predictions that were not included in the first or second computational predictions. As expected from the design of the prediction model, more compounds different from known drugs were predicted than the first or second computational prediction. Some predicted compounds were more similar to T5853872 (Fig. 5C) than known drugs or compounds in the additional data. This finding suggests influence of the feedback of our second experiment.

References

C.-C. Chang and C.-J. Lin. *LIBSVM: a library for support vector machines*, 2001. Software available at `http://www.csie.ntu.edu.tw/~cjlin/libsvm`.

F. Chen, G.A. Rodan, and A. Schmidt. Development of selective androgen receptor modulators and their therapeutic applications. *Zhonghua Nan Ke Xue*, 8:162–168, 2002.

D. Compagno, C. Merle, A. Morin, C. Gilbert, J.R. Mathieu, A. Bozec, C. Mauduit, M. Benahmed, and F. Cabon. SIRNA-directed in vivo silencing of androgen receptor inhibits the growth of castration-resistant prostate carcinomas. *PLoS ONE*, 2:e1006, 2007.

P.S. Danielian, R. White, J.A. Less, and M.G. Parker. Identification of a conserved region required for hormone dependent transcriptional activation by steroid hormone receptors. *EMBO J.*, 11:1025–1033, 1992.

J.W. Funder and J.E. Mercer. Cimetidine, a histamine H2 receptor antagonist, occupies androgen receptors. *J. Clin. Endocrinol. Metab.*, 48:189–191, 1979.

W. Gao, P.J. Reiser, C.C. Coss, M.A. Phelps, J.D. Kearbey, D.D. Miller, and J.T. Dalton. Selective androgen receptor modulator treatment improves muscle strength and body composition and prevents bone loss in orchidectomized rats. *Endocrinology*, 146:4887–4897, 2005.

I. Kinoyama, N. Taniguchi, A. Toyoshima, E. Nozawa, T. Kamikubo, M. Imamura, M. Matsuhisa, K. Samizu, E. Kawanimani, T. Niimi, N. Hamada, H. Koutok, T. Furutani, M. Kudoh, M. Okada, M. Ohta, and S. Tsukamoto. (+)-(2R,5S)-4-[4-cyano-3-(trifluoromethyl)phenyl]-2,5-dimethyl-N-[6-(trifluoromethyl)pyridin-3-yl]piperazine-1-carboxamide (YM580) as an orally potent peripherally selective nonsteroidal androgen receptor antagonist. *J. Med. Chem.*, 49:716–726, 2006.

J.T. Link, B. Sorensen, J. Patel, M. Grynfarb, A. Goos-Nilsson, J. Wang, S. Fung, D. Wilcox, B. Zinker, P. Nguyen, B. Hickman, J.M. Schmidt, S. Swanson, Z. Tian, T.J. Reisch, G. Rotert, J. Du, B. Lane, T.W. von Geldern, and P.B. Jacobson. Antidiabetic activity of passive nonsteroidal glucocorticoid receptor modulators. *J. Med. Chem.*, 48:5295–5304, 2005.

N. Nagamine and Y. Sakakibara. Statistical prediction of protein-chemical interactions based on chemical structure and mass spectrometry data. *Bioinformatics*, 23:2004–2012, 2007.

Y. Okuno, A. Tamon, H. Yabuuchi, S. Niijima, K. Tomoura, and C. Feng. GLIDA: GPCR–ligand database for chemical genomics drug discovery and tools update. *Nucleic Acids Res.*, 36:D907–912, 2008.

J. Platt. Probabilistic outputs for support vector machines and comparison to regularized likelihood methods. In A. Smola, P. Bartlett, B. Schölkopf, and D. Schuurmans, editors, *Advances in Large Margin Classifiers*, pages 61–74. MIT Press, Cambridge, MA, USA, 2000.

B.L. Roth, E. Lopez, S. Patel, and W.K. Kroeze. The Multiplicity of Serotonin Receptors: Uselessly Diverse Molecules or an Embarrassment of Riches? *The Neuroscientist*, 6:252–262, 2000.

B. Scholkopf, J.C. Platt, J. Shawe-Taylor, A.J. Smola, and R.C. Williamson. Estimating the support of a high-dimensional distribution. *Neural Computation*, 13:1443–1471, 2001.

V. Vapnik. *Statistical Learning Theory.* Wiley, New York, USA, 1998.

W. N. Venables and B. D. Ripley. *Modern Applied Statistics with S.* Springer, New York, fourth edition, 2002. URL `http://www.stats.ox.ac.uk/pub/MASS4`. ISBN 0-387-95457-0.

C. Wang, C. Ding, R.F. Meraz, and S.R. Holbrook. PSoL: a positive sample only learning algorithm for finding non-coding RNA genes. *Bioinformatics*, 22:2590–2596, 2006.

D.S. Wishart, C. Knox, A.C. Guo, D. Cheng, S. Shrivastava, D. Tzur, B. Gautam, and M. Hassanali. DrugBank: a knowledgebase for drugs, drug actions and drug targets. *Nucleic Acids Res.*, pages D901–D906, 2008.

Y. Xue, Z.R. Li, C.W. Yap, L.Z. Sun, X. Chen, and Y.Z. Chen. Effect of molecular descriptor feature selection in support vector machine classification of pharmacokinetic and toxicological properties of chemical agents. *J. Chem. Inf. Comput. Sci.*, 44:1630–1638, 2004.

Z. Zhu, R.R. Becklin, D.M. Desideio, and J.T. Dalton. Mass spectrometric characterization of the human androgen receptor ligand-binding domain expressed in Escherichia coli. *Biochemistry*, 40:10756–10763, 2001.
