# Supplementary material for: Integrating Statistical Predictions and Experimental Verifications for Enhancing Protein-Chemical Interaction Predictions in Virtual Screening
Source: PLoS Comput Biol. 2009 Jun 5;5(6):e1000397. doi: 10.1371/journal.pcbi.1000397 (PMC2685987; doi:10.1371/journal.pcbi.1000397)
Supplement: Figure S2 — Protein-drug interaction network for several datasets. (0.38 MB PDF) [file pcbi.1000397.s003.pdf]

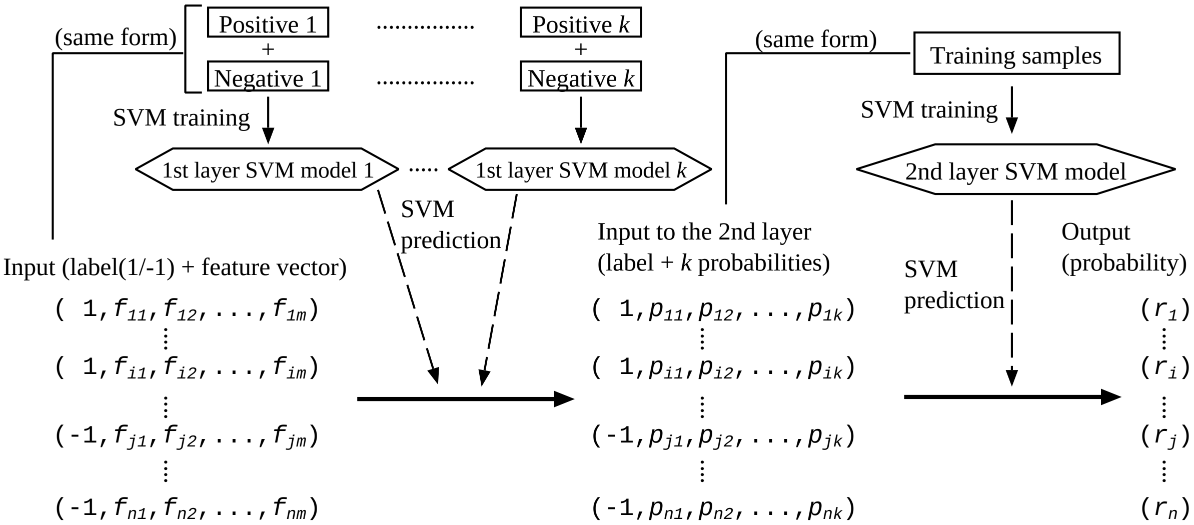

Fig. S1 Schematic illustration of the two-layer SVM system. It functions as follows.

1. Given $\boldsymbol{\mathcal{X}} = \{\boldsymbol{x}_1 = (f_{11}, f_{12}, \ldots, f_{1l}), \boldsymbol{x}_2 = (f_{21}, f_{22}, \ldots, f_{2l}), \ldots, \boldsymbol{x}_n = (f_{n1}, f_{n2}, \ldots, f_{nl})\}$, where f_{ij} is the value for the jth dimension of the feature vector for the sample, or the protein-chemical pair i, which is expressed on the basis of Eqs (3) and (4) in Supplementary Materials.
2. k first-layer SVM models are applied to $\boldsymbol{\mathcal{X}}$ to produce $\boldsymbol{\mathcal{P}} = \{\boldsymbol{P}_1 = (p_{11}, p_{12}, \ldots, p_{1k}), \boldsymbol{P}_2 = (p_{21}, p_{22}, \ldots, p_{2k}), \ldots, \boldsymbol{P}_n = (p_{n1}, p_{n2}, \ldots, p_{nk})\}$, where p_{ij} is the output of the first-layer SVM model j applied to the sample i and shows the possibility that i is positive and that is calculated on the basis of Eq. (2) in Supplementary Materials.
3. The second-layer SVM model is applied to $\boldsymbol{\mathcal{P}}$ to produce the final output $\boldsymbol{\mathcal{R}} = (r_1, r_2, \ldots, r_n)$, where r_i is the possibility that the sample i is positive.
