# Supplementary material for: Integrating Statistical Predictions and Experimental Verifications for Enhancing Protein-Chemical Interaction Predictions in Virtual Screening
Source: PLoS Comput Biol. 2009 Jun 5;5(6):e1000397. doi: 10.1371/journal.pcbi.1000397 (PMC2685987; doi:10.1371/journal.pcbi.1000397)
Supplement: Figure S3 — Effects of feature selection on two-layer SVM model. (0.02 MB PDF) [file pcbi.1000397.s004.pdf]

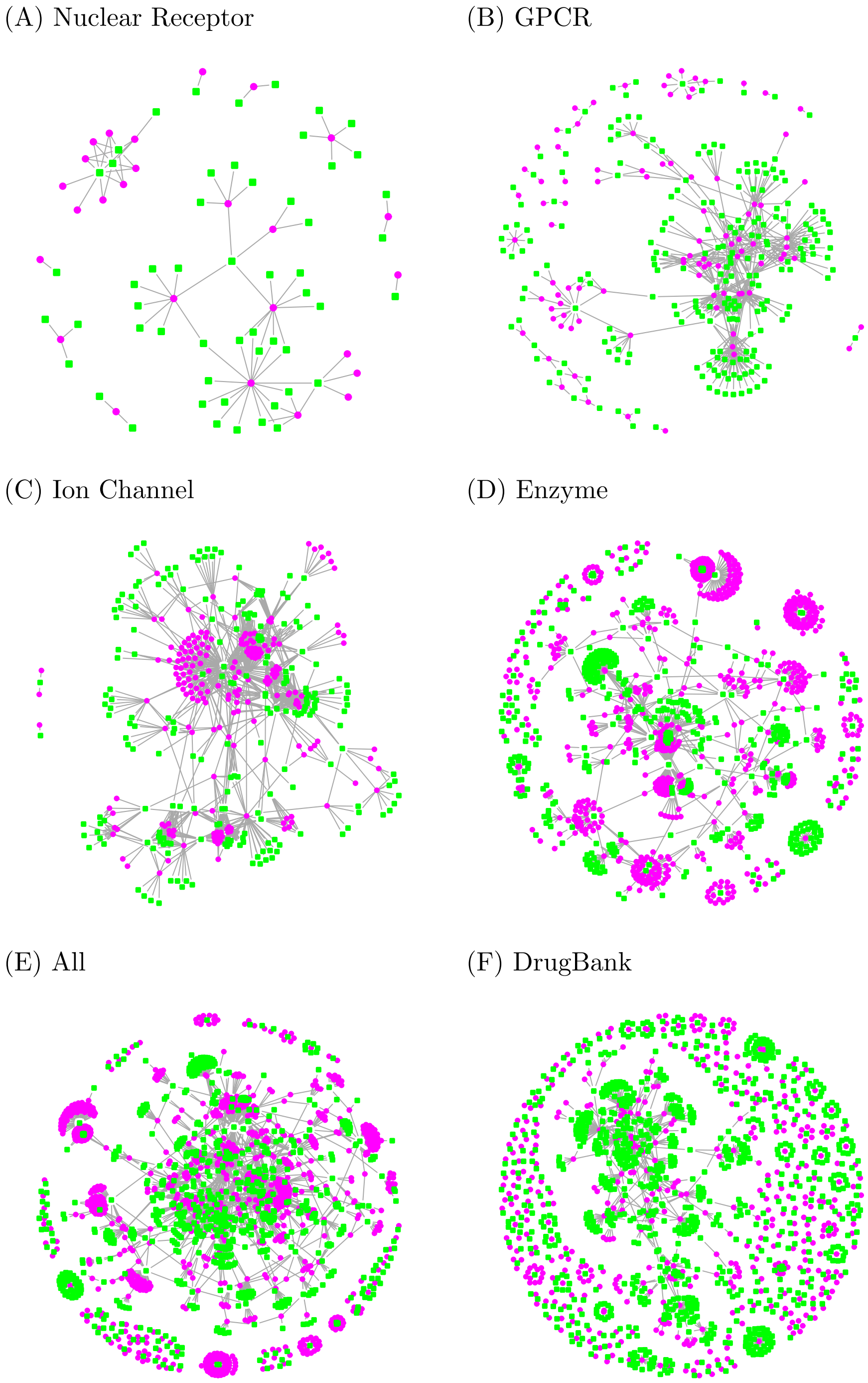

Fig. S2 Protein-drug interaction network for several datasets. A magenta circle represents a protein and a green square corresponds to a chemical compound. A gray edge means that a drug binds to a protein.
