# Supplementary material for: Integrating Statistical Predictions and Experimental Verifications for Enhancing Protein-Chemical Interaction Predictions in Virtual Screening
Source: PLoS Comput Biol. 2009 Jun 5;5(6):e1000397. doi: 10.1371/journal.pcbi.1000397 (PMC2685987; doi:10.1371/journal.pcbi.1000397)
Supplement: Figure S4 — Results of in vitro binding assay. Results of in vitro binding assay for each compound. (0.62 MB PDF) [file pcbi.1000397.s005.pdf]

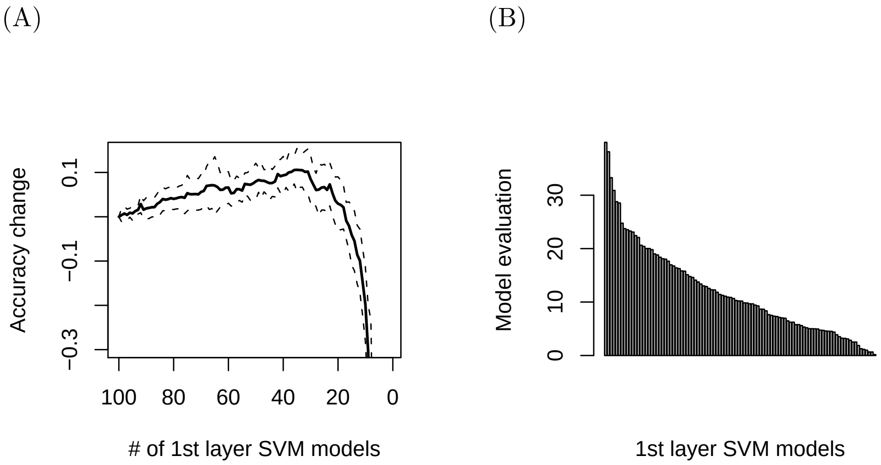

Fig. S3 Effects of feature selection on two-layer SVM model. (A) Accuracy change according to the number of *subpos* first-layer SVM models used to generate the two-layer SVM model. The accuracy obtained by using 100 fist layer SVM models (Sec. 1.2 in Supplementary Materials) and 10-fold cross-validation is set for zero, which corresponds to more than 99% accuracy. The solid line shows the mean of 12 two-layer SVM models. Two dotted lines mean 95% confidence intervals on the assumption of normal distribution. (B) Evaluation of first-layer SVM models based on the Eq. (7) in Supplementary Materials.
