# Supplementary material for: Integrating Statistical Predictions and Experimental Verifications for Enhancing Protein-Chemical Interaction Predictions in Virtual Screening
Source: PLoS Comput Biol. 2009 Jun 5;5(6):e1000397. doi: 10.1371/journal.pcbi.1000397 (PMC2685987; doi:10.1371/journal.pcbi.1000397)
Supplement: Figure S5 — The scope of the third computational prediction. (0.01 MB PDF) [file pcbi.1000397.s006.pdf]

A.

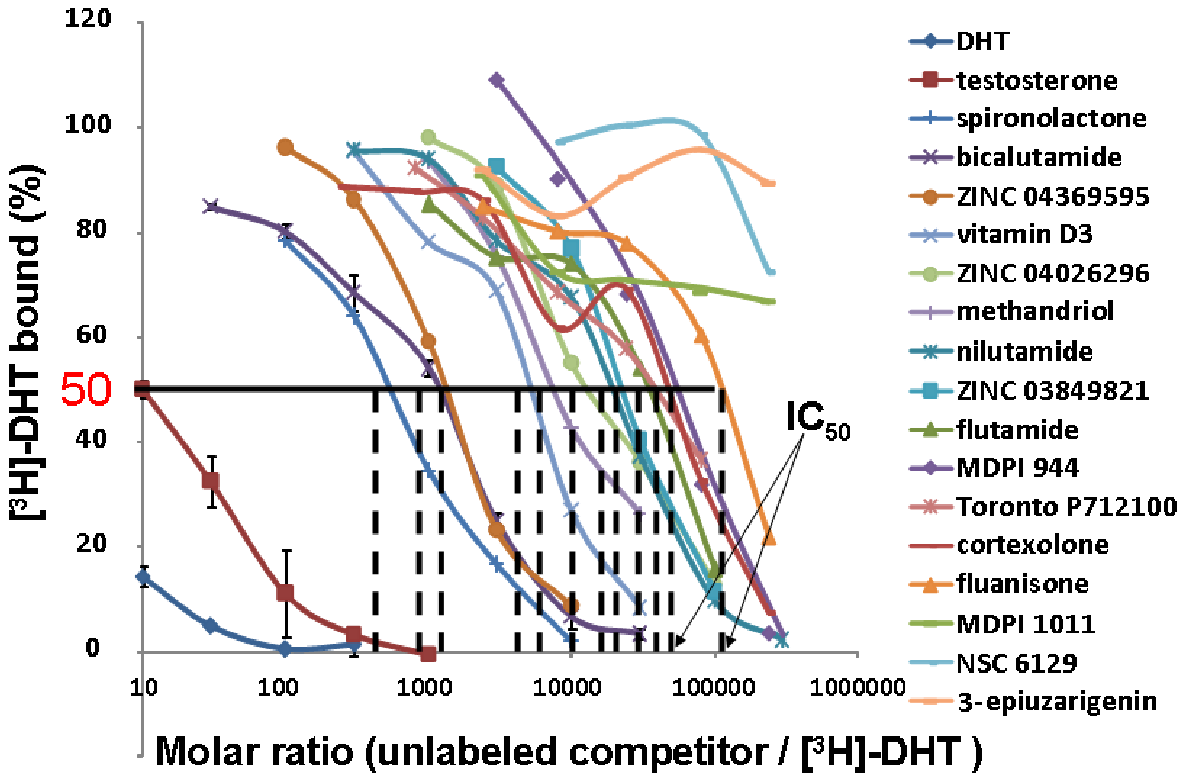

Fig. S4 A: results of the first *in vitro* binding assay.

(First experimental verification)

I. *In vitro* binding assay of DHT and testosterone

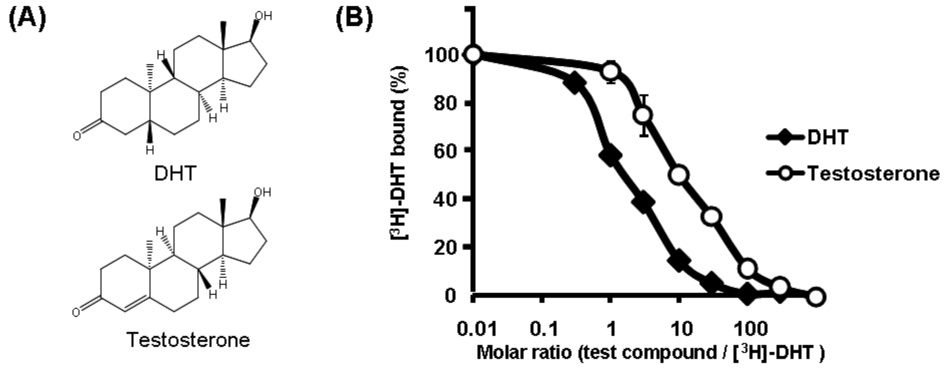

(A) chemical structure of dihydrotestosterone (DHT) and testosterone. (B) result of *in vitro* assay.

II. *In vitro* binding assay of flutamide

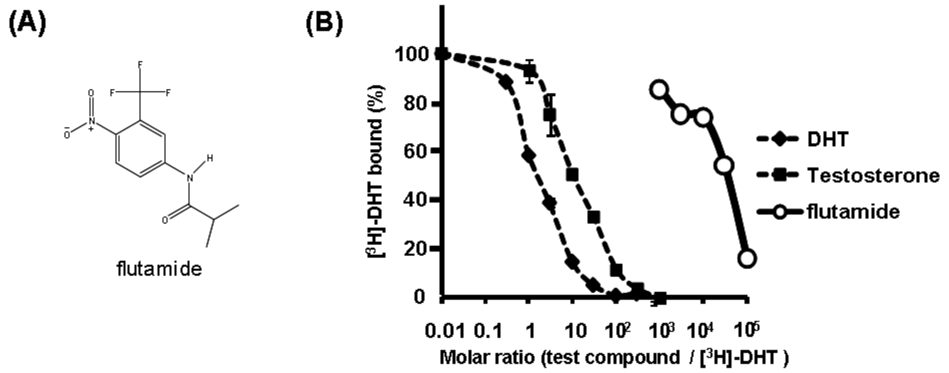

(A) chemical structure of flutamide. (B) result of *in vitro assay.*

III. *In vitro* binding assay of bicalutamide

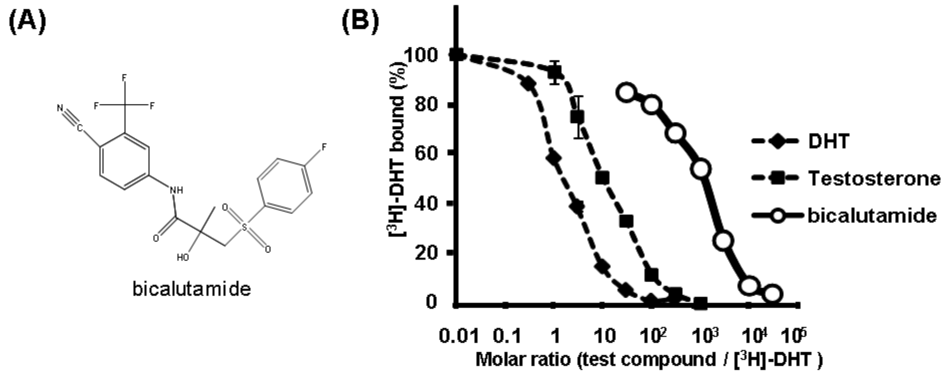

(A) chemical structure of bicalutamide. (B) result of *in vitro assay.*

IV. *In vitro* binding assay of nilutamide

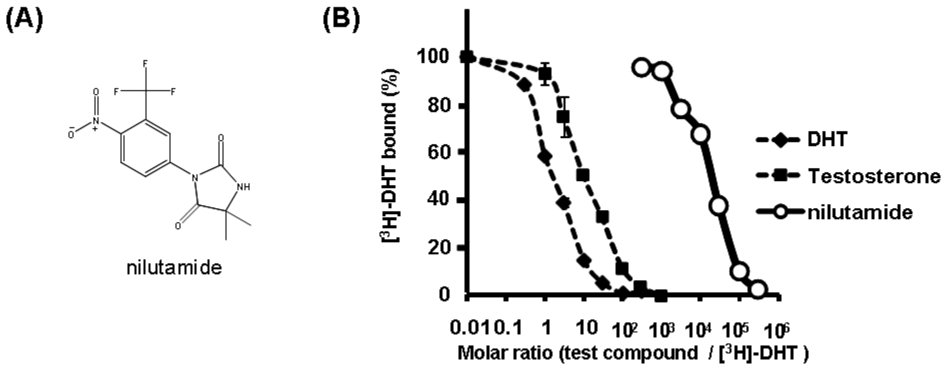

(A) chemical structure of nilutamide. (B) result of *in vitro assay.*

V. *In vitro* binding assay of spironolactone

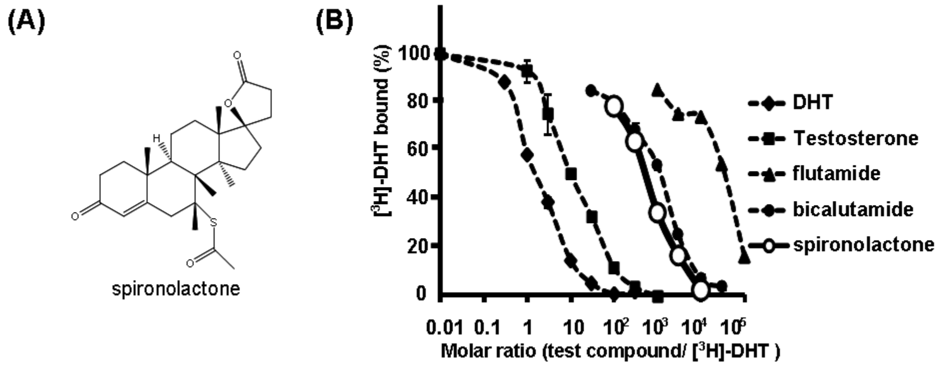

(A) chemical structure of spironolactone. (B) result of *in vitro assay.*

VI. *In vitro* binding assay of vitamin D3

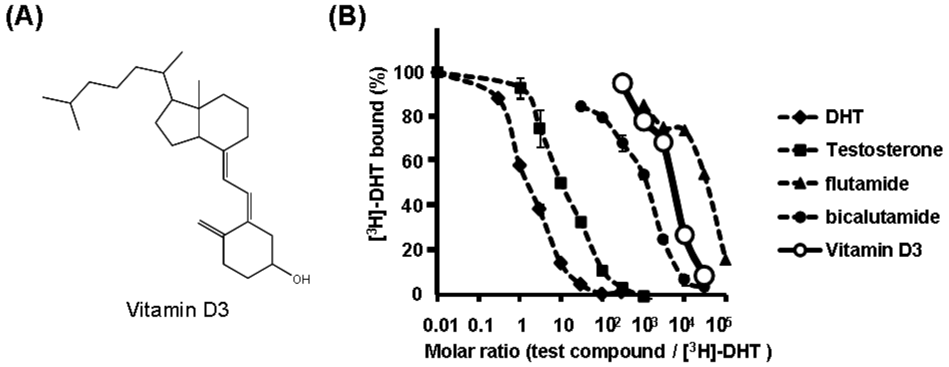

(A) chemical structure of vitamin D3. (B) result of *in vitro assay.*

VII. *In vitro* binding assay of ZINC 03849821

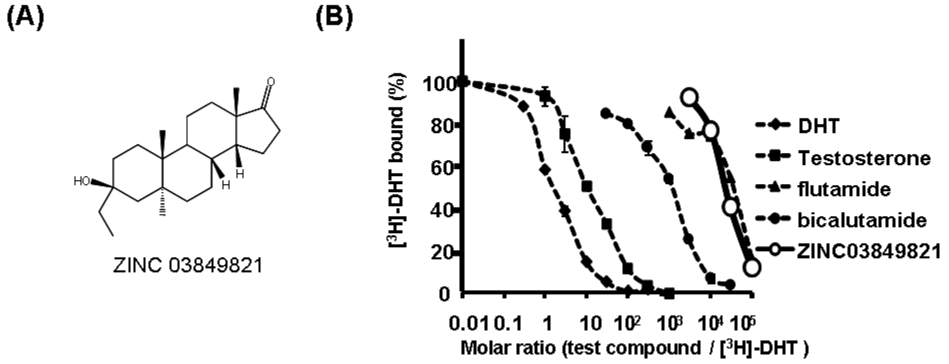

(A) chemical structure of ZINC 03849821. (B) result of *in vitro assay.*

VIII. *In vitro* binding assay of ZINC 04369595

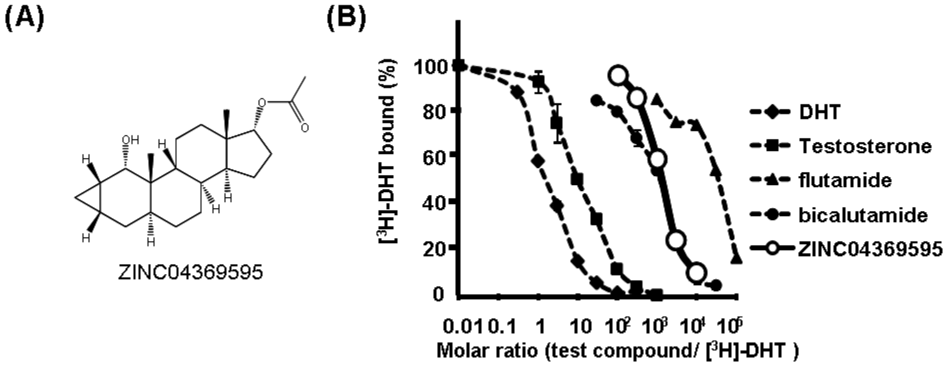

(A) chemical structure of ZINC 04369595. (B) result of *in vitro assay.*

IX. *In vitro* binding assay of fluanisone

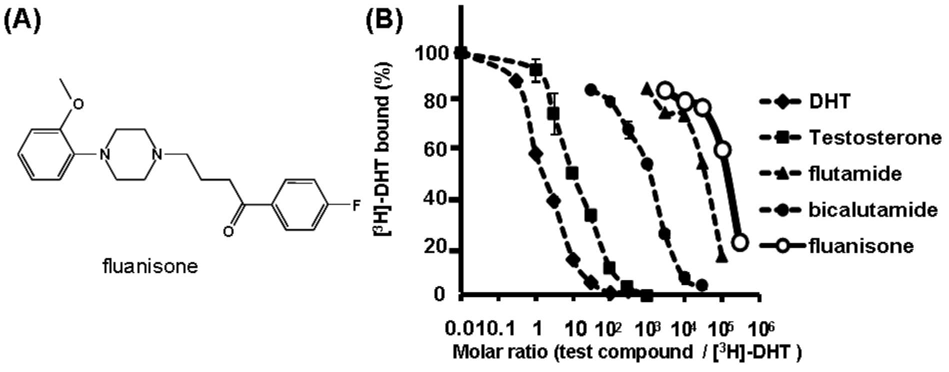

(A) chemical structure of fluanisone. (B) result of *in vitro assay.*

X. *In vitro* binding assay of ZINC 04026296

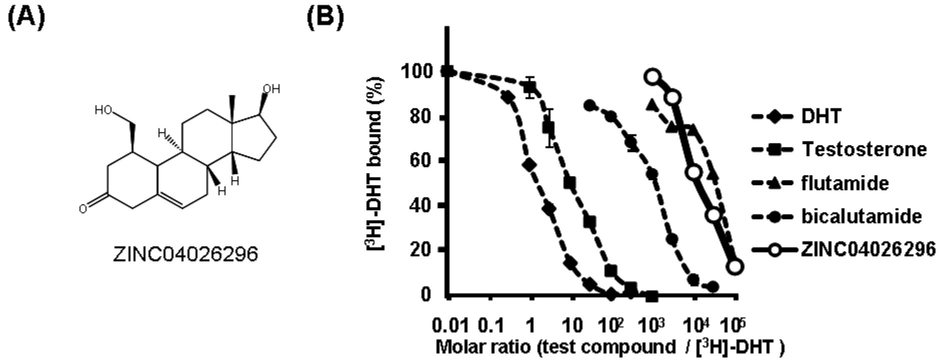

(A) chemical structure of ZINC 04026296. (B) result of *in vitro assay.*

XI. *In vitro* binding assay of 3-epiuzarigenin

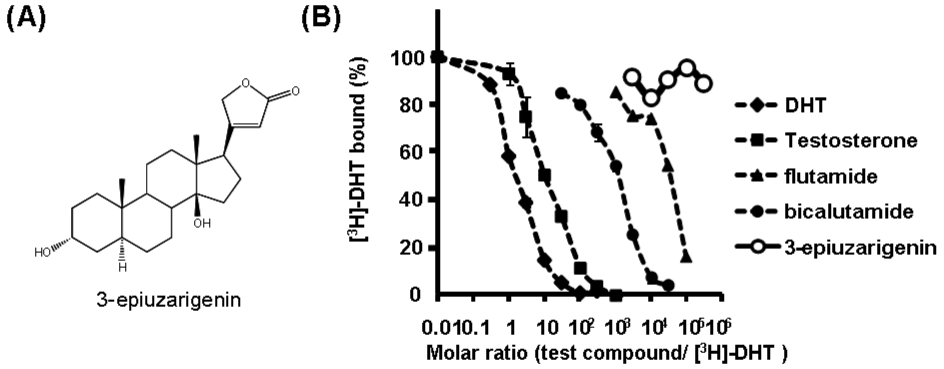

(A) chemical structure of 3-epiuzarigenin. (B) result of *in vitro assay.*

XII. *In vitro* binding assay of MDPI 1011

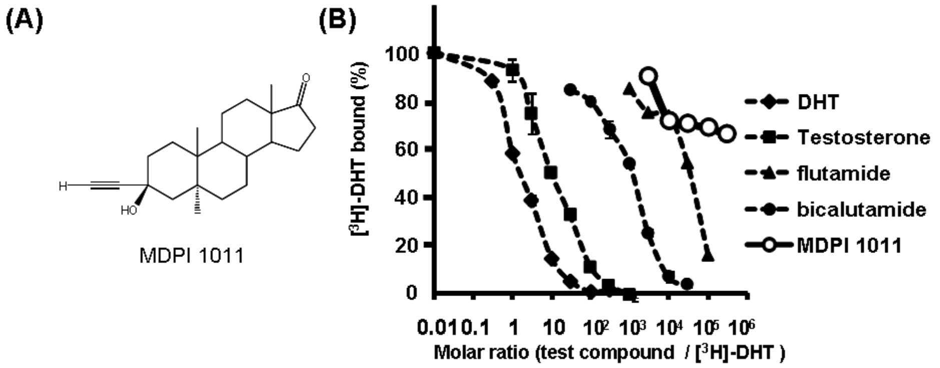

(A) chemical structure of MDPI 1011. (B) result of *in vitro assay.*

XIII. *In vitro* binding assay of NSC 6129

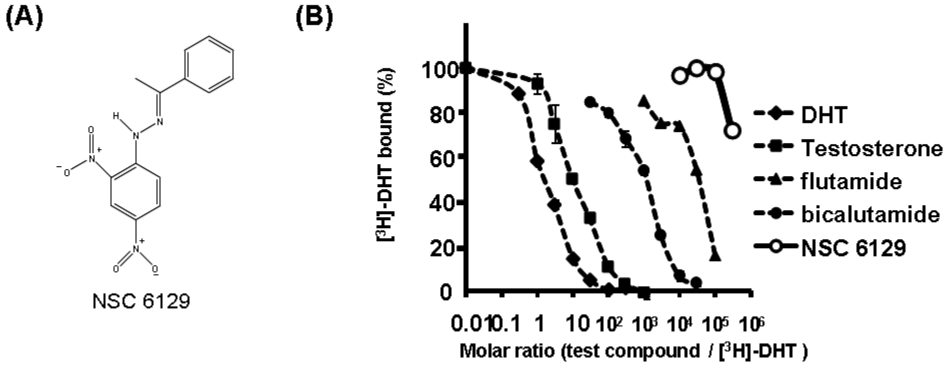

(A) chemical structure of NSC 6129. (B) result of *in vitro assay.*

XIV. *In vitro* binding assay of MDPI 944

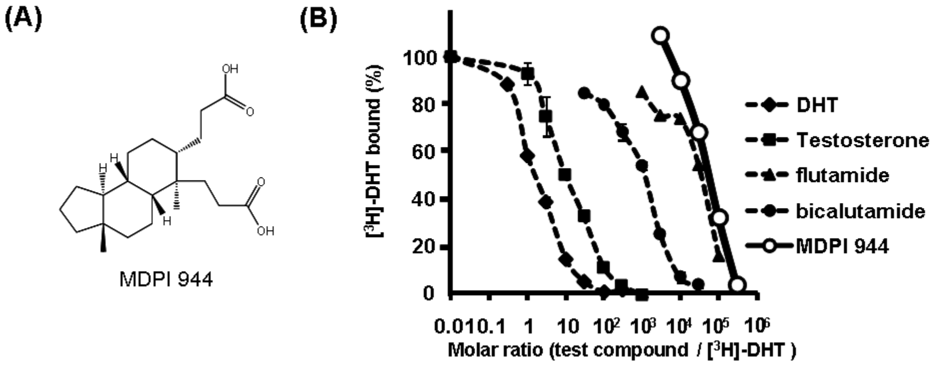

(A) chemical structure of MDPI 944. (B) result of *in vitro assay.*

XV. *In vitro* binding assay of P712100

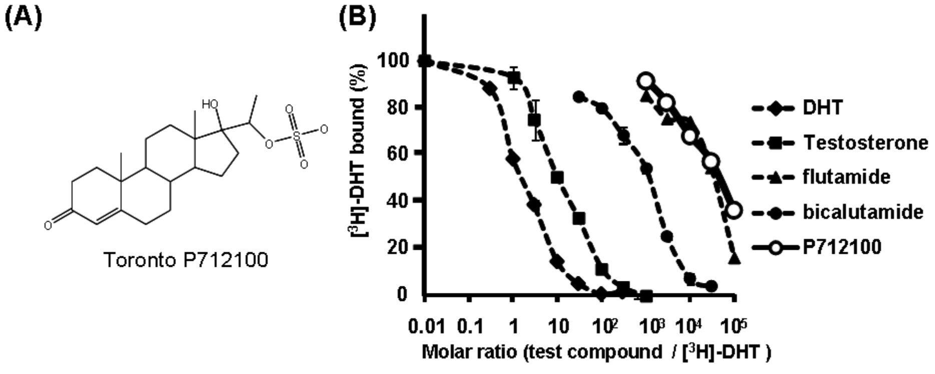

(A) chemical structure of P712100. (B) result of *in vitro assay.*

XVI. *In vitro* binding assay of methandriol

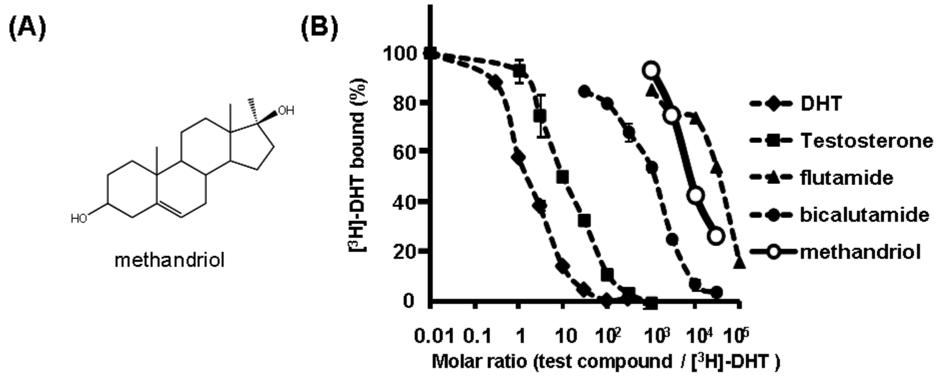

(A) chemical structure of methandriol. (B) result of *in vitro assay.*

XVII. *In vitro* binding assay of cortexolone

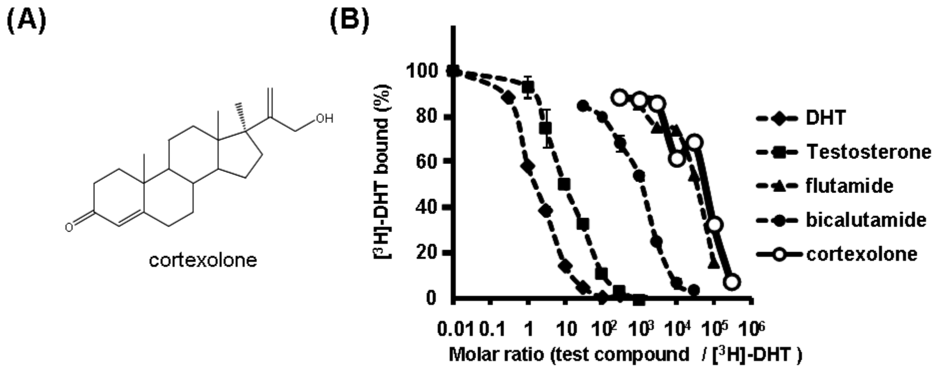

(A) chemical structure of cortexolone. (B) result of *in vitro assay.*

(Second experimental verification)

XVIII. *In vitro* binding assay of DSHS00507SC

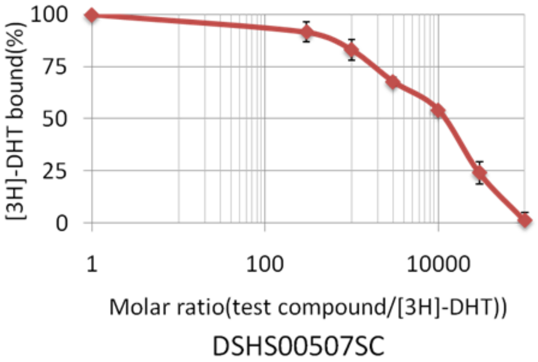

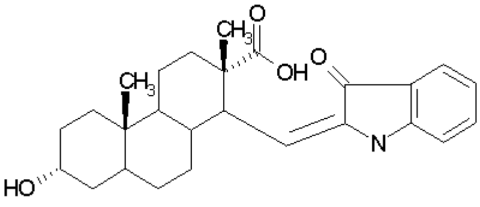

IC_{50} 22 μM

XIX. *In vitro* binding assay of 4J-584S

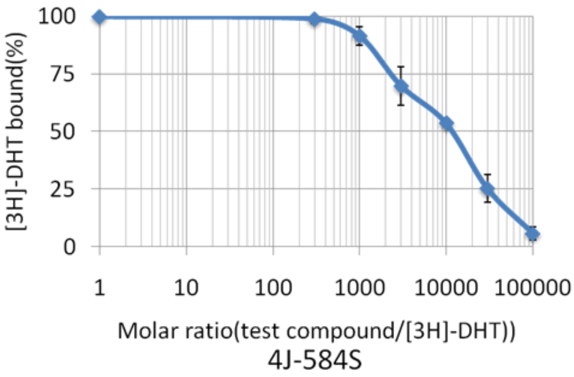

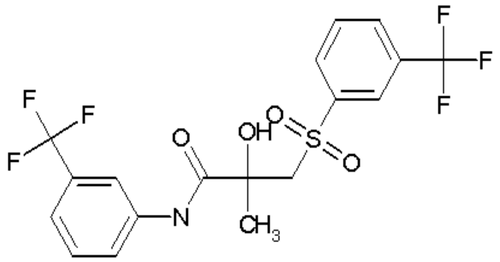

IC_{50} 22 μM

XX. *In vitro* binding assay of T5853872

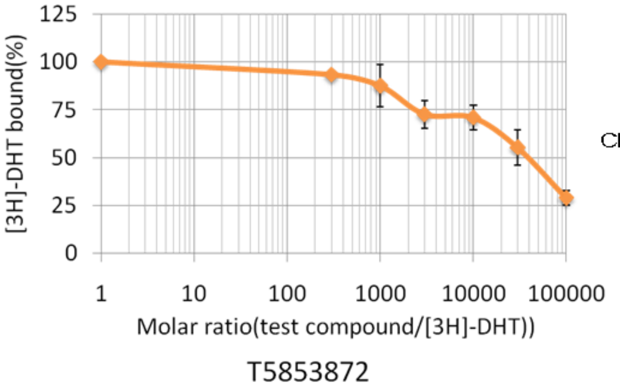

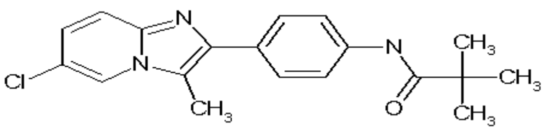

IC_{50} 80 μM

XXI. *In vitro* binding assay of BAS01279920

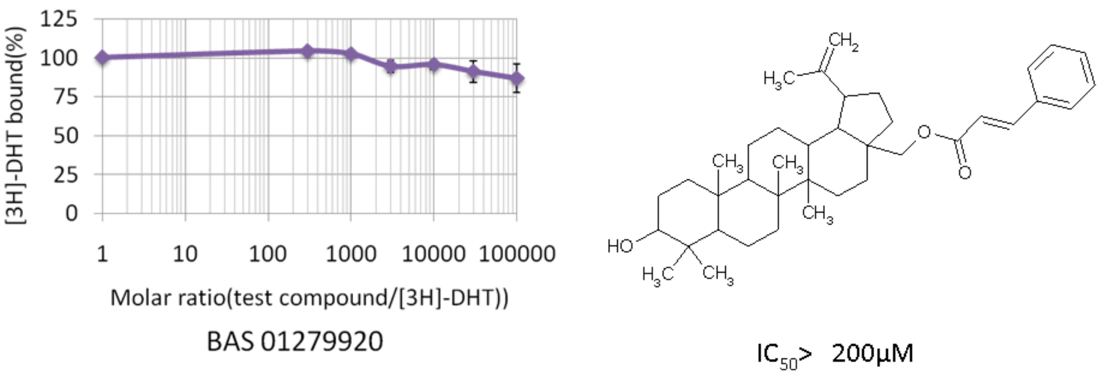

XXII. *In vitro* binding assay of AN-652/43163258

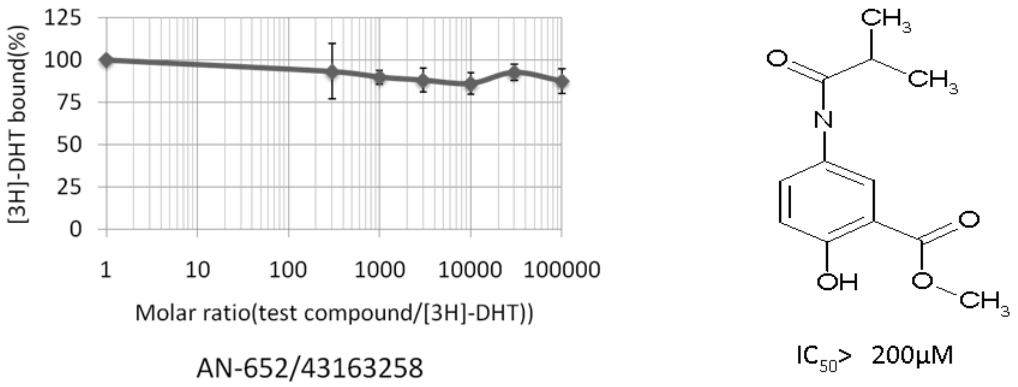

Fig. S4 I-XXII: result of *in vitro* binding assay for each compound.
