# Supplementary material for: Integrating Statistical Predictions and Experimental Verifications for Enhancing Protein-Chemical Interaction Predictions in Virtual Screening
Source: PLoS Comput Biol. 2009 Jun 5;5(6):e1000397. doi: 10.1371/journal.pcbi.1000397 (PMC2685987; doi:10.1371/journal.pcbi.1000397)
Supplement: Table S1 — Prediction performances in several datasets (0.03 MB PDF) [file pcbi.1000397.s007.pdf]

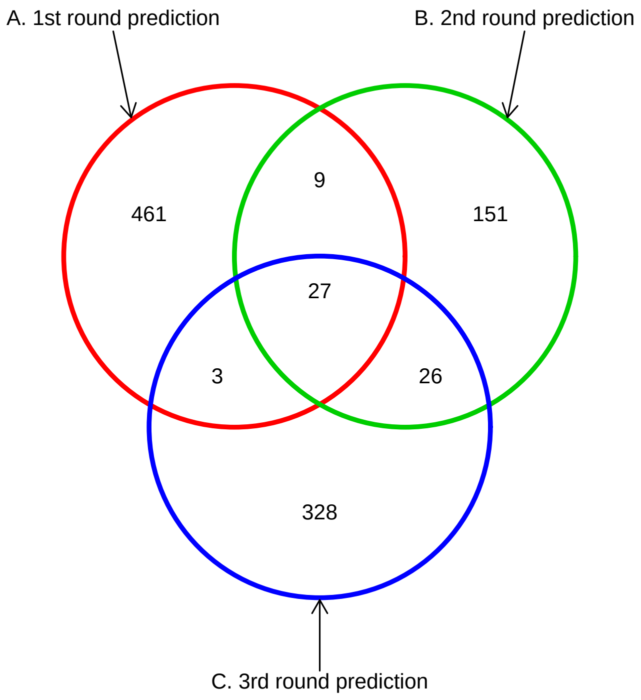

Fig. S5 The scope of the third computational prediction. A. 500 predictions made with *model B* in Fig. 4B. B. 213 predictions made with *model D* in Fig. 4D. C. 384 predictions made with a model (= *model E*) in which the results of our second experiment were utilized and pairs between chemical compounds with structure similar to steroid and AR were treated as negatives in constructing the additional model and the second-layer SVM model. There were some compounds like vitamin D3 (Fig. S4-VI) that were treated as positives in *model D* but as negative in *model E*. The weighting factor $w = 10$ was used for all the additional models.
