# Supplementary material for: Integrating Statistical Predictions and Experimental Verifications for Enhancing Protein-Chemical Interaction Predictions in Virtual Screening
Source: PLoS Comput Biol. 2009 Jun 5;5(6):e1000397. doi: 10.1371/journal.pcbi.1000397 (PMC2685987; doi:10.1371/journal.pcbi.1000397)
Supplement: Table S2 — Effects of integrating different types of protein-chemical interactions (0.02 MB PDF) [file pcbi.1000397.s008.pdf]

Table S1 Prediction performances in several datasets

dataset	Reference	# of proteins	# of drugs	# of positives	# of negatives
DrugBank	Suppl. Fig. S2F, Wishart *et al.*, 2008	456	964	1731	3500
Nuclear Receptor	Suppl. Fig. S2A, Yamanishi *et al.*, 2008	26	54	90	200
GPCR	Suppl. Fig. S2B, Yamanishi *et al.*, 2008	95	223	635	1300
Ion Channel	Suppl. Fig. S2C, Yamanishi *et al.*, 2008	204	210	1476	3000
Enzyme	Suppl. Fig. S2D, Yamanishi *et al.*, 2008	664	445	2926	5900
All	Suppl. Fig. S2E, Yamanishi *et al.*, 2008	989	791	5127	10300

dataset	[1] AUC	[2] sensitivity (%)	[3] precision (%)	[4] specificity (%)	[5] accuracy (%)
DrugBank	0.882	70.1	82.2	92.5	85.1
Nuclear Receptor	0.862	54.4	77.8	93.0	81.0
GPCR	0.906	75.3	81.6	91.7	86.3
Ion Channel	0.943	85.3	85.9	93.1	90.5
Enzyme	0.951	84.2	88.3	94.5	91.1
All	0.956	85.2	88.3	94.4	91.3

dataset	* AUC	* sensitivity (%)	* precision (%)	* specificity (%)	* accuracy (%)
DrugBank	0.877	72.3	78.6	90.3	84.3
Nuclear Receptor	0.841	57.8	70.3	89.0	79.3
GPCR	0.893	73.4	77.8	89.8	84.4
Ion Channel	0.931	83.3	82.2	91.1	88.6
Enzyme	0.943	83.1	85.2	92.8	89.6
All	0.948	84.0	85.5	92.9	90.0

[1]: area under the ROC curve.
[2]: sensitivity $= TP/(TP + FN)$.
[3]: precision $= TP/(TP + FP)$.
[4]: specificity $= TN/(TN + FP)$.
[5]: accuracy $= (TP + TN)/(TP + FN + TN + FP)$.
(TP: a number of known positives predicted as positive. FP: a number of negatives predicted as positive. FN: a number of known positives predicted as negative. TN: a number of negatives predicted as negative.)
*: RBF kernel was applied to the feature vector concatenating that of protein and that of chemical compound. The results suggest that consideration of combination effects (Eq.(5) in Supplementary Materials) generally improves prediction performances.

References

D.S. Wishart, C. Knox, A.C. Guo, D. Cheng, S. Shrivastava, D. Tzur, B. Gautam, and M. Hassanali. DrugBank: a knowledgebase for drugs, drug actions and drug targets. *Nucleic Acids Res.*, pages D901–D906, 2008.

Y. Yamanishi, M. Araki, A. Gutteridge, W. Honda, and M. Kanehisa. Prediction of drug-target interaction networks from the integration of chemical and genomic spaces. *Bioinformatics*, pages i232–240, 2008.
