# Supplementary material for: Integrating Statistical Predictions and Experimental Verifications for Enhancing Protein-Chemical Interaction Predictions in Virtual Screening
Source: PLoS Comput Biol. 2009 Jun 5;5(6):e1000397. doi: 10.1371/journal.pcbi.1000397 (PMC2685987; doi:10.1371/journal.pcbi.1000397)
Supplement: Table S3 — Prediction performances on different designed negatives (0.02 MB PDF) [file pcbi.1000397.s009.pdf]

Table S2 Effects of integrating different types of protein-chemical interactions

[2]training dataset	Prediction performances on test sets[1]			
	[3]AUC	[4]precision (%)	[5]sensitivity (%)	[6]accuracy (%)
NR	0.589 (±0.053)	100 (±0.0)	16.7 (±12.8)	58.3 (±6.4)
NR + GPCR	0.695 (±0.049)	100 (±0.0)	25.0 (± 9.0)	62.5 (±4.5)
NR + IC	0.765 (±0.020)	100 (±0.0)	20.0 (±12.0)	60.0 (±6.0)
NR + GPCR + IC	0.774 (±0.040)	100 (±0.0)	23.3 (±12.0)	61.7 (±6.0)

[1]: The four test sets consisted of 30 positive and 30 negative pairs of nuclear receptors and drugs. Here, a 'positive' pair refers to a pair of a target protein and a drug binding to the protein, and a 'negative' pair refers to a pair of a target protein and a drug which does not bind to the protein but to other proteins. Averages and 95% confidence intervals are shown.
[2]: The NR (Nuclear Receptor) training dataset consisted of 60 positive pairs of nuclear receptors and their drugs, as well as 1,284 negatives. The GPCR training dataset consisted of 635 positive pairs of GPCRs and their drugs, as well as 650 designed negatives. The IC (Ion Channel) training dataset consisted of 1,476 positive pairs of their ion channels and drugs, as well as 1,500 designed negatives. Details regarding the positive pairs are provided in Table S1.
[3]: area under the ROC curve.
[4]: precision $= TP/(TP + FP)$.
[5]: sensitivity $= TP/(TP + FN)$.
[6]: accuracy $= (TP + TN)/(TP + FN + TN + FP)$.
(TP: a number of known positives predicted as positive. FP: a number of negatives predicted as positive. FN: a number of known positives predicted as negative. TN: a number of negatives predicted as negative.)
