# Supplementary material for: Integrating Statistical Predictions and Experimental Verifications for Enhancing Protein-Chemical Interaction Predictions in Virtual Screening
Source: PLoS Comput Biol. 2009 Jun 5;5(6):e1000397. doi: 10.1371/journal.pcbi.1000397 (PMC2685987; doi:10.1371/journal.pcbi.1000397)
Supplement: Table S4 — Evaluation of our prediction method on an external test set (0.06 MB PDF) [file pcbi.1000397.s010.pdf]

Table S3 Prediction performances on different designed negatives

[1]dataset type	[2]sensitivity(%)	[3]precision (%)	[4]accuracy (%)	[5]AUC	[6]MCC
min	94.51	96.69	99.42	0.9916	0.9521
mlt	88.68	96.42	99.04	0.9941	0.9189
mle	89.08	96.01	99.04	0.9948	0.9192
max	80.47	88.05	97.99	0.9831	0.8329

[1]: refers to negative data expansion rules described in Sec. 1.3 in Supplementary Materials. 10 *subpos* first-layer SVM models were utilized to produce the two-layer SVM model from datasets with 24,500 negatives.
[2]: sensitivity $= TP/(TP + FN)$.
[3]: precision $= TP/(TP + FP)$.
[4]: accuracy $= (TP + TN)/(TP + FN + TN + FP)$.
[5]: area under the ROC curve.
[6]:$(TP \times TN - FP \times FN)/\sqrt{(TP + FN) \times (TN + FP) \times (TP + FP) \times (TN + FN)}$.
(TP: a number of known positives predicted as positive. FP: a number of negatives predicted as positive. FN: a number of known positives predicted as negative. TN: a number of negatives predicted as negative.)
