# Supplementary material for: Integrating Statistical Predictions and Experimental Verifications for Enhancing Protein-Chemical Interaction Predictions in Virtual Screening
Source: PLoS Comput Biol. 2009 Jun 5;5(6):e1000397. doi: 10.1371/journal.pcbi.1000397 (PMC2685987; doi:10.1371/journal.pcbi.1000397)
Supplement: Table S5 — Evaluation of our method with respect to comprehensive interaction prediction (0.01 MB PDF) [file pcbi.1000397.s011.pdf]

Table S4 Evaluation of our prediction method on an external test set

[2]Model type	[1]Internal 10-fold cross-validation<br>[3]prec.(%)	 [4]sens.(%)	 [5]acc.(%)	[1]External prediction<br>prec.(%)	 sens.(%)	 acc.(%)
(A) Effect of rational negative design						
one-layer	71.76	42.99	95.11	64.66	50.59	95.00
one-layer$_r$	82.38(±0.64)	38.22(±0.95)	95.38(±0.06)	40.68(±1.19)	50.00(±1.87)	92.02(±0.28)
(B) Effect of second-layer SVM model						
subpos	97.11	92.57	99.33	82.81	31.18	95.11
subpos$_r$	94.40(±0.67)	96.46(±1.00)	99.39(±0.11)	42.5(±2.71)	33.53(±3.90)	92.74(±0.41)
subpos$_{\mathrm{v}_{0.5}}$	–	–	–	8.89	57.06	59.27
subpos$_{\mathrm{v}_{0.8}}$	–	–	–	28.13	5.29	92.98
subpos$_{\mathrm{m}_{0.5}}$	–	–	–	9.52	58.24	61.37
subpos$_{\mathrm{m}_{0.8}}$	–	–	–	17.86	2.94	92.82
subpos$_{\mathrm{ann}}$	95.98	93.21	99.29	75.81	27.65	94.73
subpos$_{\mathrm{qda}}$	70.69	54.39	95.49	34.52	17.06	92.52
subpos$_f$	95.66(±0.32)	78.33(±1.60)	98.33(±0.10)	78.76(±2.86)	25.59(±1.09)	94.71(±0.09)
(C) Improvement of precision						
allpos	99.68	100.00	99.98	100.00	10.59	94.20
subpos$_{\lfloor 0.9 \rfloor}$	–	–	–	90.70	22.94	94.85
subpos$_{\lfloor 0.95 \rfloor}$	–	–	–	92.50	21.76	94.81
one-layer$_{\lfloor 0.9 \rfloor}$	–	–	–	86.67	15.29	94.35
one layer$_{\lfloor 0.95 \rfloor}$	–	–	–	71.43	2.941	93.63

[1]: The external dataset consisted of 170 positives and 2,450 negatives that were randomly chosen from 1,731 positives and 24,500 designed negatives with the *mlt* rule described in Sec. 1.3 in Supplementary Materials and that were excluded in constructing first-layer and second-layer SVM models. Here, “one layer” SVM model was produced based on the same features as used for the first-layer SVM model. The internal dataset utilized 1,561 positives and 22,050 negatives.
[2]: "Model type" exhibits that the one-layer SVM model or the second-layer SVM model, specified by the type of first-layer SVM model, was utilized. Subscripts mean as follows.

- $_r$ means that three types of randomly chosen 22,050 pairs of protein and chemical compound were used instead of designed negatives to construct the SVM model. The 95% confidence intervals are shown.
- $_{\mathrm{v}_t}$ means that voting with 11 first-layer SVM models with threshold t was used for prediction.

$$pred_{\mathrm{v}_t} = \begin{cases} \text{pos.} & \text{if } \sum_{i=1}^{11} r_i \geq 6 \\ \text{neg.} & \text{otherwise} \end{cases}, \quad r_i = \begin{cases} 1 & \text{if (probability output of model } i) \geq t \\ 0 & \text{otherwise} \end{cases}$$

- $_{\mathrm{m}_t}$ means that average of outputs of 11 first-layer SVM models was used for prediction with threshold t.

$$pred_{\mathrm{m}_t} = \begin{cases} \text{pos.} & \text{if } \sum_{i=1}^{11} \frac{\text{(probability output of model } i)}{11} \geq t \\ \text{neg.} & \text{otherwise} \end{cases}$$

- $_{\mathrm{ann}}$ means that Artificial Neural Network (ann) (implemented by the statistical software package R (`http://cran.r-project.org/`) function *nnet* (Venables and Ripley, 2002)) was applied to outputs of 11 first-layer SVM models. Parameters were selected to give the best accuracy in the internal 10-fold cross validation. For example, 17 units were used in the hidden layer.
- $_{\mathrm{qda}}$ means that Quadratic Discriminant Analysis (qda) (implemented by R function *qda* (Venables and Ripley, 2002)) was applied to outputs of 11 first-layer SVM models.
- $_f$ means that twenty types of randomly chosen 11 first-layer SVM models were used to construct the second-layer SVM model. The 95% confidence intervals are shown.
- $_{\lfloor t \rfloor}$ (e.g. $t = 0.9$) means that final probability outputs were evaluated with threshold t.

[3]: precision (prec.) $= TP/(TP + FP)$.
[4]: sensitivity (sens.) $= TP/(TP + FN)$.
[5]: accuracy (acc.) $= (TP + TN)/(TP + FN + TN + FP)$.
(TP: a number of known positives predicted as positive. FP: a number of negatives predicted as positive. FN: a number of known positives predicted as negative. TN: a number of negatives predicted as negative.)

References

W. N. Venables and B. D. Ripley. *Modern Applied Statistics with S*. Springer, New York, fourth edition, 2002. URL `http://www.stats.ox.ac.uk/pub/MASS4`. ISBN 0-387-95457-0.
