# Supplementary material for: Integrating Statistical Predictions and Experimental Verifications for Enhancing Protein-Chemical Interaction Predictions in Virtual Screening
Source: PLoS Comput Biol. 2009 Jun 5;5(6):e1000397. doi: 10.1371/journal.pcbi.1000397 (PMC2685987; doi:10.1371/journal.pcbi.1000397)
Supplement: Table S6 — Utilization of one-class SVM in the selection of negative samples (0.03 MB PDF) [file pcbi.1000397.s012.pdf]

Table S6 Utilization of one-class SVM in the selection of negative samples

(A) One-class SVM models constructed from the DrugBank dataset.

[1]kernel	10-fold cross validation accuracy (%)	[2]self-prediction accuracy
sigmoid	87.2	92.1
RBF	50.1	50.0
polynomial	50.0	50.0

(B) Results of comprehensive prediction

[3]kernel	[4]rule	[5]accuracy	[6]P10275	[6]P11229	[6]P35367	[7]evaluation
RBF	*min*	99.9	81317	40897	35466	27.2
RBF	*mle*	99.9	4470	10977	22240	61.7
sigmoid	*min*	99.8	79553	54180	48262	11.9
sigmoid	*mle*	97.8	16194	3316	392	13.5

[1]: Kernel functions used to train one-class SVM models. RBF ($f(\boldsymbol{x}, \boldsymbol{y} = \exp(-\gamma|\boldsymbol{x} - \boldsymbol{y}|^2))$, sigmoid ($f(\boldsymbol{x}, \boldsymbol{y}) = \tanh(\gamma \boldsymbol{x}^t \boldsymbol{y} + c)$), and polynomial ($f(\boldsymbol{x}, \boldsymbol{y}) = (\gamma \boldsymbol{x}^t \boldsymbol{y} + c)^d$). Here, γ, c and d are constants.

[2]: prediction accuracy when a prediction model was constructed from the whole DrugBank dataset and applied to the DrugBank dataset.

[3]: kernel functions used to train the second-layer SVM model.

[4]: selection rule of candidates for negative data. The one-class SVM model using the sigmoid kernel (A) was applied to all the combinations of proteins and chemical compounds in DrugBank dataset except positives, which were represented as described in Sec. 2.3 in Supplementary Materials, and negative samples were selected according to the following selection rules.

min: Top n samples in the ascending order of the decision values.

mle: Top n samples in the descending order of the decision values whose decision values were under 0.

Here, if the decision value for a sample was above 0, the sample was supposed to be included in the high-density region which was estimated from positive samples. There existed no samples whose decision value equaled to 0.

[5]: 10-fold cross-validation accuracy ($= (TP + TN)/(TP + FN + TN + FP)$) of the second-layer SVM. 24,500 negatives and 10 *subpos* first-layer SVM models were used to train the second-layer SVM model. TP: true positives, TN: true negatives, FN: false negatives, FP: false positives.

[6]: target proteins whose ligands were predicted on the basis of 109,841 compounds. The number of predicted binding compounds is shown.

[7]:

$$\text{evaluation} = 100 \times \left(\frac{1}{2} \left[\text{rec}_{0.5} + \frac{\text{rec}_{0.95} + \text{prec}_{0.95}}{2\{1 + (1 - \text{rec}_{0.95})(1 - \text{prec}_{0.95})\}} \right] - \frac{\text{total \# of predicted positives - \# of known positives}}{\text{total \# of prediction targets - \# of known positives}} \right),$$

where rec_x is the recall rate (=TP/(TP+FN)) at the threshold x, ranging from 0 to 1. 0.5 is the threshold following the definition of SVM. TP: true positives, FN: false negatives.
