# Supplementary material for: Integrating Statistical Predictions and Experimental Verifications for Enhancing Protein-Chemical Interaction Predictions in Virtual Screening
Source: PLoS Comput Biol. 2009 Jun 5;5(6):e1000397. doi: 10.1371/journal.pcbi.1000397 (PMC2685987; doi:10.1371/journal.pcbi.1000397)
Supplement: Table S7 — Overlaps of predictions between prediction models in Table S5 (0.03 MB PDF) [file pcbi.1000397.s013.pdf]

Table S7 Overlaps of predictions between prediction models in Table S5

(A) threshold= 0.5

	1.	2.	3.	4.	5.
1. [1]similarity	5265	1161	1145	654	354
2. only compound	–	3269	2007	658	345
3. [2]one-layer	–	–	3309	[†] 895	388
4. [3]*subpos*	–	–	–	1163	309
5. [4]*allpos*	–	–	–	–	388

(B) threshold= 0.95

	1.	2.	3.	4.	5.
1. similarity	5265	359	260	337	253
2. only compound	–	456	171	200	176
3. one-layer	–	–	268	175	200
4. *subpos*	–	–	–	428	179
5. *allpos*	–	–	–	–	265

[†]: There were 895 compounds common between 3,309 compounds predicted by the one-layer SVM model and 1,163 obtained by the *subpos* two-layer SVM model.
[1]: A chemical compound i is predicted as a binding ligand of a protein α by the similarity method if

$$pred_{\mathrm{sim}}(i) = \max_{j \in A} \frac{|I \cap J|}{|I \cup J|} \geq 0.9,$$

where A is known binding ligands of the protein α, and I (or J) is a set of substructures in $\mathcal{P}_2^6(i)$ (or $\mathcal{P}_2^6(j)$) described in Eq. (4) in Supplementary Materials.
[2]: one-layer SVM using the *mlt* dataset with 28,000 negatives.
[3]: two-layer SVM using 10 *subpos* first-layer SVM models and the *mlt* dataset with 24,500 negatives .
[4]: two-layer SVM using 9 *allpos* first-layer SVM models and the *max* dataset with 28,000 negatives.
[*]: details of *mlt* and *max* datasets are provided in Sec. 1.3 in Supplementary Materials.
